# GWOA: A multi-strategy enhanced whale optimization algorithm for engineering design optimization

**DOI:** 10.1371/journal.pone.0322494

**Published:** 2025-09-03

**Authors:** Yanzhao Gu, Junhao Wei, Zikun Li, Baili Lu, Shirou Pan, Ngai Cheong

**Affiliations:** 1 Faculty of Applied Sciences, Macao Polytechnic University, Macao 999078, China; 2 College of Animal Science and Technology, Zhongkai University of Agriculture and Engineering, Guangzhou 510225, China; 3 School of Economics and Management, South China Normal University, Guangzhou 510006, China; Torrens University Australia, AUSTRALIA

## Abstract

This paper analyzes the shortcomings of the traditional Whale Optimization Algorithm (WOA), mainly including the tendency to fall into local optima, slow convergence speed, and insufficient global search ability for high-dimensional and complex optimization problems. An improved Whale Optimization Algorithm (GWOA) is proposed to overcome these issues. By integrating several improvement strategies, such as adaptive parameter adjustment, enhanced prey encircling, and sine-cosine search strategies, GWOA significantly enhances global search ability and convergence efficiency. However, GWOA increases computational complexity, which may lead to longer computation times when handling large-scale problems. It may also fall into local optima in high-dimensional cases. Several experiments were conducted to verify the effectiveness of GWOA. First, 23 classic benchmark functions were tested, covering unimodal, multimodal, and compositional optimization problems. GWOA was compared with other basic metaheuristic algorithms, excellent WOA variants, and the latest algorithms. Then, a comparative scalability experiment is performed on GWOA. The experimental results showed that GWOA achieved better convergence speed and solution accuracy than other algorithms in most test functions, especially in multimodal and compositional optimization problems, with an Overall Efficiency (OE) value of 74.46%. In engineering optimization problems, such as pressure vessel design and spring design, GWOA effectively reduced costs and met constraints, demonstrating stronger stability and optimization ability. In conclusion, GWOA significantly improves the global search ability, convergence speed, and solution stability through multi-strategy integration. It shows great potential in solving complex optimization problems and provides an efficient tool for engineering optimization applications.

## 1 Introduction

Metaheuristic algorithms are computational methods used to solve optimization problems, especially large-scale, complex, and high-dimensional ones. Unlike traditional exact algorithms, metaheuristic algorithms do not rely on a specific mathematical model or gradient information of the objective function. Instead, the problem is solved by mimicking the ’heuristics’ of natural or social phenomena. Metaheuristic algorithms have powerful global search capabilities, exploring a wide range of solutions to avoid local optima. The core idea is to design an efficient, practical algorithm that provides high-quality solutions in most cases [1]. Among these high-quality solutions, some may approach the optimal solution. Although there is no guarantee of optimality, it is possible to find approximate optimal solutions in a large search space. Thus, these algorithms are able to find a balance between global and local search. Combining stochasticity with heuristic strategies leads to improved solution efficiency and optimization performance. Metaheuristic algorithms are widely used in a variety of optimization problems, including those with irregular, discrete, or noisy characteristics. There are many metaheuristic algorithms, with typical examples including the Genetic Algorithm (GA) [2], Artificial Bee Colony (ABC) [3], Particle Swarm Optimization (PSO) [4], Dung Beetle Optimization (DBO) [5], Grey Wolf Optimizer (GWO) [6], Harris Hawk Optimization (HHO) [7], Sine-Cosine Algorithm (SCA) [8], Whale Optimization Algorithm (WOA) [9], and others. When solving real-world problems, both modeling and optimization are usually required. Modeling evaluates the objective function by using a correct mathematical model, while optimization is used to achieve the optimal configuration of the design parameters. Therefore, the key to optimization is the algorithm itself. Based on this, this paper will focus on the improvement of optimization algorithms.

Metaheuristic algorithms originated in the 1970s. The earliest algorithms, such as Simulated Annealing (SA) [10] and GA, , were inspired by nature’s annealing process and evolution. They have powerful global search capabilities but have large computational costs. From the 1990s to the 2000s, population-based algorithms like Particle Swarm Optimization (PSO) and Ant Colony Optimization (ACO) [11] were proposed and gradually applied to dynamic optimization problems. In the 2010s, with the rise of deep learning and big data. The newer algorithms such as Gray Wolf Optimization (GWO), Bat Algorithm (BA) [12] and Elk herd optimizer (EHO) [13] emerged. The application of hybrid algorithms has also increased, improving the performance of the algorithms in the search space. Currently, researchers are focused on improving newer algorithms and experimenting with hybrid approaches. Chen *et al*. [14] proposed a method for trajectory planning and time optimization of woodworking manipulators using 3-5-3 segmented polynomial interpolation and a modified Particle Swarm Optimization algorithm (GoldS-PSO). The method significantly improves operational efficiency, stability and smoothness in motion control. Yang *et al*. proposed a structural optimization method for lightweight adhesive modules using simulation, alternative models, and the Dung Beetle Optimizer (DBO) [15]. A weight reduction of 11.7% was achieved while maintaining adhesive stability and load capacity. Nadimi-Shahraki *et al*. proposed an Improved Gray Wolf Optimization (I-GWO) algorithm [16]. The problems of traditional GWO algorithm in terms of population diversity, local and global search balance, and premature convergence were effectively solved by introducing a dimensional learning based hunting strategy (DLH). Wang *et al*. proposed an improved hybrid hawk optimization algorithm (IHAOHHO), which enhances the global exploration and local exploitation capabilities of the AO and HHO algorithms by combining nonlinear escape energy parameters and stochastic dyadic learning strategies [17]. Tawhid *et al*.proposed a new and efficient multi-objective optimization algorithm, The Multi Objective Sine Cosine Algorithm (MO-SCA), which obtains different levels of non-domination and maintains the diversity of solution sets through elite non-domination ranking and congestion distance methods [18]. Chakraborty *et al*. proposed an improved Whale Optimization Algorithm (WOAmM) by combining the mutual benefit stage in the symbiotic organism search algorithm with WOA [19]. The problem of premature convergence is mitigated so that the search space can be explored more efficiently and avoid the overuse of computational resources. Wei *et al*. proposed an Adaptive Position Updating PSO (IPSO), which integrates inertia weight and adaptive position update strategy to improve the convergence speed of PSO and avoid premature convergence [20]. However, hybrid optimization algorithms risk overfitting, as they may overly focus on task-specific engineering constraints. This can result in good performance on specific tasks but poor generalization. Additionally, hybrid algorithms often require more computational resources, which can lead to imbalanced resource utilization in large-scale problems.

The Whale Optimization Algorithm (WOA) studied in this paper is a metaheuristic optimization algorithm based on group behavior. It is inspired by the unique feeding behavior of humpback whales and was proposed by S. Mirjalili *et al*. in 2016 [21]. Its core is to achieve the global optimization objective by simulating the predatory behavior of humpback whales, including encircling the prey, spiraling around the prey, and searching for it. WOA is widely used in function optimization, engineering problems, and image processing due to its simplicity and high efficiency. However, it often gets trapped in local optima when dealing with complex problems. Especially in large-scale search space, the algorithm may not be able to effectively jump out of the local optimal point, resulting in unsatisfactory final results. Additionally, WOA converges slowly in some cases, especially for high-dimensional problems. Its convergence is often slower than that of other optimization methods such as Particle Swarm Optimization (PSO) or Genetic Algorithms (GA). As iterations progress, the population diversity in WOA may gradually diminish, limiting its ability to explore potentially better solutions. This affects the optimization results. The balance between exploration and convergence in the algorithm is also somewhat problematic, although it performs a broader global search during the initial stages. However, as the iteration progresses, premature convergence tends to limit the algorithm’s ability to continue discovering better solutions. For high-dimensional problems, WOA’s performance is notably worse. Its performance is notably worse for high-dimensional problems due to low search efficiency and high time complexity. To address these limitations, several WOA variants have been proposed. For example, in 2019, Chen *et al*. proposed an improved whale optimization algorithm (BWOA) by introducing Lévy flight (LF) and a chaotic local search strategy (CLS) into the whale optimization algorithm (WOA), improving the balance between the global exploration capability and the local search capability of the traditional WOA. It was validated on classical engineering design optimization problems such as tension/compression springs, pressure vessel design, and three-bar truss design [22]. In 2021, Chakraborty *et al*. proposed an improved WOAmM algorithm by combining the reciprocity phase in the Symbiotic Organisms Search (SOS) with the Whale Optimization Algorithm (WOA) [19]. This enables it to enhance the exploration of the search space, thus avoiding the waste of computational resources due to overexploitation. In 2022, Yang *et al*. proposed the Multi-Strategy Whale Optimization Algorithm (MSWOA) by introducing chaotic mapping, adaptive weights, Lévy flight mechanism, and evolutionary population dynamics mechanism, and combining it with the Semi-Supervised Extreme Learning Machine (SSELM) [23]. The problems of the whale optimization algorithm, which is prone to falling into local optimal solutions and experiencing slow convergence, are solved. Optimizing the parameter selection significantly improves the classification accuracy and performance in engineering applications. In the same year, Chakraborty *et al*. proposed an Improved Whale Optimization Algorithm (ImWOA) by adjusting the random selection process in the search for prey phase, and introducing a cooperative hunting strategy for whales [24]. It divides its iterative process into two phases: exploration and exploitation. exploration and exploitation. Thus, it increases the diversity of the solution, avoids local optimal solutions, and improves the accuracy and convergence speed of the solution. However, the variants of algorithms proposed by researchers generally increase the difficulty of algorithm design, implementation, and parameter tuning. Since each variant has different hyperparameters, selecting and adjusting them becomes challenging. Additionally, improper parameter settings for a specific sub-algorithm may affect the overall performance. Furthermore, the convergence of variant algorithms may become unstable, especially when the characteristics of different algorithms vary greatly. Some algorithms may converge quickly, while others may lead to local optima or slow convergence, thus reducing overall efficiency.

To address this, this paper proposes the GWOA, which accelerates the convergence process by providing a high-quality starting point through the Good Nodes Set initialization. The Growth-based Encircling Prey strategy enhances the balance and stability of the search; the Synergetic Search-for-Prey strategy prevents over-reliance on a single individual, improving the systematic nature of the search process; the Adaptive Sin-Cosine strategy effectively regulates the relationship between global and local searches, reducing the risk of local optima; and the improved Cauchy Mutation strategy based on DE further improves the diversity of the search, preventing the algorithm from getting trapped in local optima. Through the integration of these strategies, the improved GWOA demonstrates stronger robustness, global search capability, and convergence speed in various complex optimization problems. The introduction of adaptive mechanisms and dynamic adjustments reduces dependence on parameters, making the algorithm more efficient and easier to implement across various applications.

## 2 Related research on engineering design optimization challenges

Engineering optimization aims to find an optimal solution under specific constraints. Typically by minimizing or maximizing an objective function, such as cost, time, resource consumption, or performance. These problems often involve discontinuous, non-differentiable functions, non-convex surfaces, multimodal problems, or noisy functions, making them difficult to solve efficiently with traditional methods. Additionally, deterministic methods have high computational costs, especially for large solution spaces requiring exhaustive searches. In practical optimization, near-optimal solutions are generally acceptable, as engineers often prefer to quickly find a satisfactory solution rather than spend excessive time pursuing a marginally better one [25]. With advances in technology and computational power, optimization algorithms have become indispensable tools in various engineering fields. Engineering optimization problems can be classified into linear, nonlinear, integer, compositional, multi-objective, and dynamic categories. This paper focuses on enhancing the Whale Optimization Algorithm (WOA) for nonlinear engineering optimization problems, such as Pressure Vessel design [26], Gear Train design [27], Corrugated Bulkhead design [28] and Speed Reducer design [29].

In recent years, engineering design optimization of Pressure Vessels, Tension/Compression Springs, Piston Levers and Speed Reducers has shifted from traditional mathematical models to simulation-based intelligent optimization techniques. Meta-heuristic algorithms like Gray Wolf Optimization (GWO), Whale Optimization (WOA), and Particle Swarm Optimization (PSO) are widely used in these areas. These algorithms do not rely on precise mathematical models and can handle complex design spaces, performing well in multi-objective and multi-constraint optimization problems. Jun *et al*. proposed the Cauchy Gray Wolf Optimization Algorithm (CGWO), which improves convergence speed, solution accuracy, and robustness through techniques like Cauchy distribution initialization, dynamic inertia weighting, and a greedy strategy [30]. It significantly improves the convergence speed, solution accuracy and robustness in terms of global search capability, balanced exploration and exploitation, and avoidance of early convergence, and outperforms traditional methods in engineering applications. Eleonora *et al*. optimized the design of tension/compression springs using a variety of popular population intelligence algorithms aiming to minimize the weight of the springs [31]. Hu *et al*. proposed an energy-feedback suspension system that combines magnetorheological dampers (MRDs) with a whale optimization algorithm-proportional integral differential (WOA-PID) control algorithm, and demonstrated that the system possesses a certain energy recovery capability in suspension control [32]. Zhou *et al*. proposed an improved whale optimization algorithm (LWOA) based on Lévy flight [33]. By enhancing the local optimal jumping ability, its outperformance over WOA was verified on the Speed Reducer. This paper proposes an improved algorithm GWOA to address WOA’s limitations. Meanwhile, this paper will verify that GWOA outperforms the original WOA and other algorithms in terms of convergence speed, robustness, and stability in the above four engineering optimization problems.

## 3 Whale optimization algorithm

The Whale Optimization Algorithm (WOA) is a metaheuristic algorithm proposed by Mirjalili *et al*. in 2016, inspired by the hunting behavior of humpback whales [21]. The WOA simulates the spiral updating strategy and encircling prey strategy exhibited by humpback whales during predation.

### 3.1 Initialization

During initialization, the population distribution and parameter settings are defined. The population is initialized by setting the individual positions, *X*_*i*_, where each individual represents a solution and the population consists of candidate solutions. The fitness (objective function value) for each individual is then calculated, and the individual with the best fitness becomes the initial global best solution ${\text{X}}^{*}$. Each whale’s position is randomly initialized in the search space. Assuming the search space is j-dimensional, the initial position of the ${\text{i}}^{\text{th}}$ individual is given below:

$$X_{i,j}=Rand\cdot (ub-lb)+lb$$ (1)

where *ub* and *lb* are the upper and lower bounds of the decision variables; and *Rand* is a random value between 0 and 1.

### 3.2 Encircling prey

The WOA search space is the global solution space, with the prey’s position determined first. Since the location in the solution space is not known a priori, WOA assumes the current best solution is the target, and other whale individuals approach the current best solution. When |A|<1, whales perform a shrinking encircling action, gradually approaching the target and focusing on local development near the best solution. This helps improve convergence and optimization accuracy but may lead to premature trapping in local optima. The specific position update formula is as follows:

$$D=|C\cdot X^{*}(t)-X(t)|$$ (2)

$$X(t+1)=X^{*}(t)-A\cdot D$$ (3)

where ${\text{X}}^{*}$ is the current global best solution; *X* is the current whale position; and *A* and *C* are coefficients used to control the shrinkage and exploration, influencing the attraction or repulsion of whales to the prey. These coefficients are calculated as follows:

A=2a·r−a (4)

C=2·r (5)

$$a=2-2\cdot \frac{t}{T}$$ (6)

where parameter *a* starts at 2 and linearly decreases to 0 with iterations; *r* is a random value between [0, 1]; *T* is the maximum number of iterations; and *t* is the current iteration.

### 3.3 Bubble-net attacking method

Spiral updating is a unique bubble-net hunting behavior of humpback whales, which gradually approach the prey by spiraling around it. It is a local exploration strategy. When p≥ 0.5, the spiral updating formula is as follows:

$$D^{*}=|X^{*}(t)-X(t)|$$ (7)

$$X(t+1)=D^{*}\cdot e^{bl}\cdot \cos(2\pi l)+X^{*}(t)$$ (8)

$$a_{1}=-1-\frac{t}{T}$$ (9)

$$l=(a_{1}-1)\cdot Rand+1$$ (10)

where ${\text{D}}^{*}$ represents the distance between the current individual position *X* and the leader ${\text{X}}^{*}$; *b* is a constant defining the shape of the spiral (usually set to 1); and the spiral coefficient *l* ranges between [–2, 1].

The exponential decay ${\text{e}}^{\text{bl}}$ and periodic movement are used to simulate the whale’s path toward the prey, while maintaining some randomness to enhance the global search ability and avoid local convergence. As the whale approaches the prey, the spiral movement becomes tighter. However, in complex search spaces, WOA’s position updates rely heavily on randomness and are entirely based on the current global best solution. This approach can be effective in the early stages but tends to converge near local optima in the later stages, struggling to escape local optima and achieve a balance between global and local search strategies.

### 3.4 Search-for-prey

When whales have not yet located the prey, to ensure all whales fully explore the solution space, one whale is randomly selected to search for the prey. This random search enlarges the exploration range and prevents trapping in local optima. When |A|≥1, the update formulas are as follows:

$$D_{rand}=|C\cdot X_{rand}-X(t)|$$ (11)

$$X(t+1)=X_{rand}-A\cdot D_{rand}$$ (12)

where *X*_*rand*_ is a random individual from the current population; and *A* controls how far the individual moves away from the current best solution to ensure global search capability.

### 3.5 Pseudo-code of WOA

The pseudo-code of the WOA is shown in Algorithm 1.

**Algorithm 1.** WOA



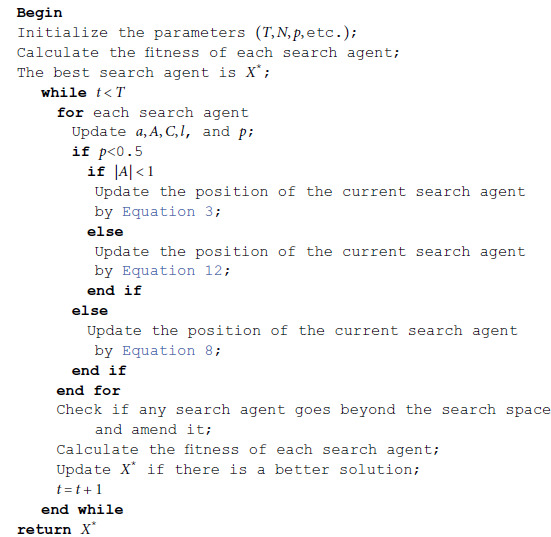



### 3.6 Advantages and disadvantages of WOA

WOA randomly initializes the population position vectors and sets the maximum number of iterations and parameter *a* firstly. Then, the global best solution is determined by evaluating the fitness. The hunting behavior of whales is simulated by updating the target position through encircling the prey and using random search strategies to select prey. The population positions are updated based on the hunting results and continue until the maximum iterations or convergence condition is met, outputting the global best solution. This algorithm approximates the optimal solution using bionics principles, offering excellent search performance and convergence. It is simple, easy to implement, and applicable to various optimization problems. Compared to complex algorithms, WOA is more computationally efficient. However, in high-dimensional, multimodal problems, the population may focus too much on local development, reducing global search capability. The algorithm’s performance is sensitive to parameter settings, and improper adjustment of parameters can reduce search effectiveness. As iterations progress, the population diversity gradually diminishes, affecting the search capability. Additionally, increasing the number of iterations increases computational costs. To address these issues, this paper introduces an enhanced whale optimization algorithm with multiple strategies, GWOA, which uses Good Nodes Set initialization to generate a uniform distribution of population, incorporates Growth-based Encircling Prey strategy with a newly-designed inertia weight ω, incorporates a novel designed Adaptive Sine-Cosine strategy, Synergetic Search-for-Prey strategy and an improved Cauchy Mutation Strategy based on DE. Meanwhile, the updating method of parameter *a* was resigned to better balance exploration and exploitation. These enhancements comprehensively improve the global search capability, convergence speed, population diversity and robustness of the WOA.

## 4 GWOA

### 4.1 Good nodes set initialization

**Fig 1:**
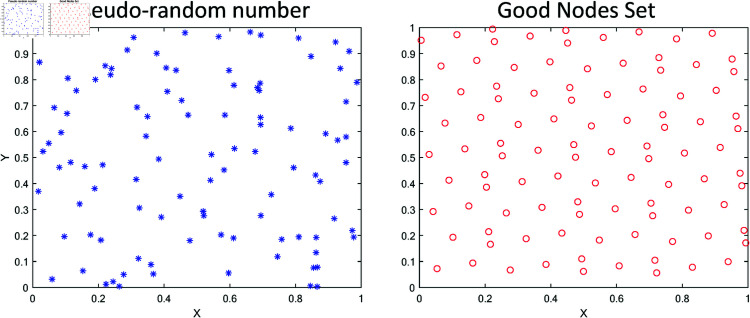
Populations generated by the initialization of the good nodes set.

The core idea behind Good Nodes Set Initialization is to select a set of high-quality initial nodes (i.e., “good nodes”) to improve global search efficiency and optimization solution quality. The basic principle is to select several excellent nodes as the initial solutions or starting nodes for the population at the beginning of the algorithm. These nodes are usually determined through heuristic methods, statistics, or other techniques, rather than being generated purely randomly. This provides the optimization algorithm with a set of starting nodes that are closer to the global optimal solution. The traditional WOA algorithm uses pseudo-random number generation to generate the population. This method is simple and exhibits strong randomness, but it also has the problem of uneven population distribution. Randomly generated populations may be overly dense in some areas and sparse in others, resulting in uneven coverage of the search space, which negatively affects the efficiency and performance of the WOA algorithm during the search process. As shown in the Fig 1, GWOA uses Good Nodes Set initialization to generate a uniformly distributed population, improving the quality of the population.

Let *D*_*n*_ be an *n*-dimensional Euclidean space’s unit cube, where there exists a point set:

$$P_{M}=(\{kr_{1}\},\{kr_{2}\},...,\{kr_{M}\}),1\leq k\leq M,r\in D_{n}$$ (13)

where *C*(*r*, ε) is a constant that depends on *r* and ε(ε > 0). Then; *P*_*M*_ is called the Good Points Set.

The value of *r* is calculated as follows:

$$r=2cos\frac{2\pi i}{p},1\leq i\leq s$$ (14)

where *p* is the smallest prime number satisfying (*p*-3)/2 ≥s . The Good Nodes Set is mapped to the actual search space using the following mapping formula:

$$P_{i}(j)=(ub_{j}-lb_{j})\cdot \{r_{j}^{i}\cdot k\}+lb_{j}$$ (15)

where *ub*_*j*_ and *lb*_*j*_ represent the upper and lower bounds of the ${\text{j}}^{\text{th}}$ dimension.

By providing high-quality initial nodes, Good Nodes Set initialization can accelerate the convergence speed of the algorithm, reduce unnecessary computations, and improve the quality of the solutions. It effectively avoids local optima issues caused by random initialization, especially in complex multi-modal optimization problems. Furthermore, it ensures uniform coverage of the search space, enhancing global search ability and preventing bias, thus improving the optimization algorithm’s performance and efficiency. This advantage is not only evident in two-dimensional spaces but also shows in high-dimensional spaces, as the construction of the Good Nodes Set itself is independent of the dimension.

### 4.2 Growth-based encircling prey

In the original prey encirclement strategy, position updates are based on a fixed linear relationship, making the search process overly dependent on the best solution. The search range remains unchanged throughout the process, which may cause the algorithm to over-explore in the early stages or fall into local optima during the exploitation phase. Shi *et al*. were the first to introduce the concept of inertia weight ω into the PSO algorithm, which led to significant performance improvement [34]. Inspired by inertia weights, this paper proposes an inertia weight update mechanism based on the Sigmoid function. The inertia weight ω is calculated as follows:

$$\omega =\frac{0.9}{1+e^{-15\left(\frac{t}{T}-0.5\right)}}$$ (16)

where ω is an inertia weight that decreases from 0.9 to 0; *t* represents the current iteration; and *T* is the maximum number of iterations.

The following are the common calculation formulas for ω:

$$\omega_{1}=0.9-0.9\cdot \sqrt{\frac{t}{T}}$$ (17)

$$\omega_{2}=0.9-0.9\cdot {\left(\frac{t}{T}\right)}^{2}$$ (18)

$$\omega_{3}=0.9-0.9\cdot \frac{t}{T}$$ (19)

**Fig 2:**
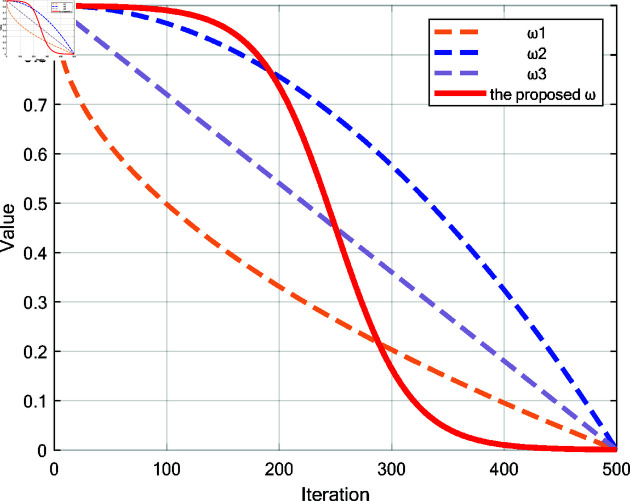
Comparison of Common ω and the Proposed ω.

As shown in the Fig 2, the major advantage of the proposed inertia weight ω lies in its smooth decrease. In the early stages, a larger ω gives the search agents strong exploration ability, allowing them to search the solution space widely. As iterations proceed, the reduction in ω reduces the dependence on the optimal solution for position updates, shifting from global exploration to local refinement. This mechanism balances exploration and exploitation at different stages, preventing premature convergence to a local optimum. Near the global optimal solution, it prevents large steps from causing excessive deviations from the optimal solution. It also makes position updates more flexible and avoids over-reliance on leaders.

The Growth-based Encircling Prey strategy is defined as follows:

$$X(t+1)=\omega \cdot X^{*}(t)-A\cdot D^{*}$$ (20)

where ω is the proposed inertia weight; ${\text{X}}^{*}$ is the current global best solution; *A* is a coefficient vector; and ${\text{D}}^{*}$ represents the distance between the current individual position *X* and the leader ${\text{X}}^{*}$, as shown in Eq 7.

### 4.3 Adaptive sine-cosine strategy

The Sine-Cosine Algorithm (SCA) is a population-based global optimization algorithm proposed by Seyedali Mirjalili [35]. It is inspired by the oscillatory behavior of the sine and cosine functions. The core idea of the SCA is to simulate the oscillatory behavior of the sine and cosine functions to find the global optimum. The algorithm is simple, easy to implement, and requires few parameters, making it suitable for various optimization problems. The optimization process of SCA is based on two main update mechanisms: the convergence property of the sine function and the oscillatory property of the cosine function. This allows SCA to explore the search space effectively and avoid local optima. To enhance the global search ability of WOA, we have introduced an adaptive sine-cosine strategy inspired by SCA. The adaptive sine-cosine strategy incorporates the oscillatory behavior of the sine function and the spiral updating behavior of the cosine function, enabling the algorithm to adaptively explore in the early stages and avoid local optima while balancing exploration and exploitation. The adaptive sine-cosine strategy is modeled as follows:

$$X(t+1)=\{\begin{array}{c} D^{*}\cdot r_{1}\cdot \sin(r_{2})\cdot |r_{3}\cdot X^{*}(t)-X(t+1)|+X^{*}(t),r<1-\sqrt{\frac{l}{T}}\\ D^{*}\cdot e^{bl}\cdot \cos(r_{4})+X^{*}(t),r\geq 1-\sqrt{\frac{l}{T}} \end{array}$$ (21)

$$r_{1}=2\cdot \left(1-\frac{t}{T}\right)$$ (22)

$$r_{2}=2\cdot \pi \cdot rand$$ (23)

$$r_{3}=2\cdot rand$$ (24)

$$r_{4}=2\cdot l\cdot \pi$$ (25)

where *r* is a random number between 0 and 1; *l* is a random number that controls the whale’s movement pattern when searching for prey, the formula is shown in Eq 10; ${\text{D}}^{*}$ is the distance between the current individual *X* and the global best solution ${\text{X}}^{*}$, the formula is shown in Eq 7; *b* is a spiral factor that adjusts the speed of the exponential function; *r*_1_ is a dynamic factor that controls the range of the sine strategy; *t* represents the current iteration; *T* is the maximum number of iterations; *r*_2_ is a random angle between 0 and 2π; *r*_3_ is a random factor between 0 and 2; and *r*_4_ controls the periodic behavior of the update position.

The adaptive sine-cosine strategy introduces sine and cosine functions to randomly adjust the search direction and uses random factor *r*_3_ to control the amplitude of the update. This provides more search dimensions and dynamic position updates, preventing the algorithm from sticking to a single hunting pattern that could cause it to get stuck in local optima. In the original WOA algorithm, the global exploration ability is strong in the early stages of a search, but as iterations progress, the search range shrinks, potentially slowing down the search speed in later stages. To address this, the Adaptive Sine-Cosine strategy introduces adaptive shrinking control parameters *r*_4_ and *r*_1_, allowing broad exploration in the early stages and gradual narrowing toward the target, reducing oscillations and deviations, which helps accelerate convergence. Additionally, the strategy uses the random factor *r* to control the selection between the sine and cosine hunting methods, allowing whale agents to perform both global and local searches. This enables dynamic control of the search phase increases the flexibility of the hunting behavior. Furthermore, the introduction of random angles and diversified range *r*_4_ enhances diversity and jumping ability, improving the chances of escaping local optima. The non-linear form of the sine-cosine strategy effectively explores the complex fitness function’s search space, improving the algorithm’s adaptability to high-dimensional problems. This strategy enhances global search capability, avoids local optima, and helps the algorithm find the optimal solution faster and more efficiently. It is especially beneficial for complex, high-dimensional optimization problems, where it can find the global optimum in fewer iterations while exhibiting stronger robustness and adaptability.

### 4.4 Synergetic search-for-prey

In the original Search-for-prey strategy, a random whale is selected each time for the search. This may cause unnecessary fluctuations in the solution space, particularly in the middle stages of the algorithm, potentially leading to an unstable search process or even jumping out of the global optimum region. The strategy is overly reliant on the randomly chosen individual, which can cause the algorithm to get stuck in local optima, especially in complex solution spaces. To reduce the excessive randomness of this strategy, we propose a new Synergetic Search-for-Prey strategy. In the Synergetic Search-for-Prey strategy, position updates are made by referencing both the global optimal solution and the average position of the current whale agents, focusing the search near the current optimal solution. This enhances local search ability and helps explore the current region more effectively and avoids unnecessary long-distance jumps. The Synergetic Search-for-Prey strategy also avoids over-reliance on a single individual, improving the diversity of the search. By introducing both the global optimal solution ${\text{X}}^{*}$ and the average position of all whale agents, and introducing a random disturbance term. The position update formula better combines the overall information of the whale population. This enables the algorithm to escape local optima, enhancing its global search ability. The Synergetic Search-for-Prey strategy is modeled as follows:

$$X(t+1)=0.5\left(X^{*}(t)+X_{M}(t)\right))-r\cdot |X^{*}(t)-2\cdot r\cdot X|$$ (26)

$$X_{R}(t)=\frac{1}{N}\sum_{i=1}^{N}X_{i}(t)$$ (27)

where *X*_*m*_ represents the average position of the whale agents, calculated in Eq 27; *r* is a random number uniformly distributed in the interval [0, 1]; *X* is the position of the current whale agent; ${\text{X}}^{*}$ is the position of the current global optimal solution.

### 4.5 Improved cauchy mutation strategy based on differential evolution

The standard position update strategy in WOA relies on biomimetic principles like prey encirclement and spiral updates. These strategies generally focus on searching near the current optimal solution, particularly in the later stages when whale agents converge on a specific area, reducing the exploration of the search space. To enhance search diversity, a mutation strategy can be introduced after position updates to perturb the current solution and help whale agents escape local optima, allowing for broader exploration. This is particularly useful when solving complex problems with multiple local optima, as the mutation strategy helps agents avoid premature convergence.

Therefore, this paper proposes an improved Cauchy Mutation Strategy based on Differential Evolution. This strategy combines the mutation mechanism of the Cauchy distribution with the differential evolution approach, enhancing the global search capability of the algorithm. It can effectively avoid stagnation near local optima and improve the exploration and convergence properties in complex optimization problems.

First, a new intermediate solution ${X_{i}^{new}}^{\prime \prime }$ is generated using the differential evolution strategy:

$${X_{i}^{new}}^{\prime \prime }=X_{i}+F\cdot (X^{*}-X_{1})+F(X_{2}-X_{3})$$ (28)

where *X*_*i*_ represents the position of the current *i*th agent; ${\text{X}}^{*}$ is the position of the current best individual; *X*_1_, *X*_2_, *X*_3_ are positions of three randomly selected different individuals from the population; and *F* is a factor controlling the scaling of the differential vector, calculated as below:

F=1+tan(π·(Rand−0.5)) (29)

where *Rand* is a random number between 0 and 1.

Next, Cauchy Mutation is applied to the intermediate solution ${X_{i}^{new}}^{\prime }$:

$$X_{i}^{new\prime }=X_{i}^{new\prime \prime }\cdot \left(1+cauchy(0,1,1,dim)\right)$$ (30)

where *cauchy*(0,1,1,*dim*) represents the perturbation generated by the Cauchy distribution.

Finally, boundary checks and adjustments are made to avoid population degradation in WOA, it as shown in Eq 31:

$$X_{i}^{new}=min(max(X_{i}^{new\prime },lb),ub)$$ (31)

If the fitness of the mutated solution $X_{i}^{new}$ is better than the original solution *X*_*i*_, then $X_{i}^{new}$ will replace *X*_*i*_.

### 4.6 The pseudo-code of GWOA

The pseudo-code of the GWOA is shown in Algorithm 2.

### 4.7 Computational complexity analysis

#### 4.7.1 Time complexity analysis

The time complexity mainly depends on the computational effort required during each iteration. We need to analyze the key steps involved in each iteration.

*Time Complexity of WOA:*

- The initialization of WOA requires generating a random matrix of size *SearchAgentsno* * *dim*, and the time complexity is O(SearchAgentsno* *dim*);
- The objective function calculation involves running the outer loop for *Maxiter* iterations. The inner loop operates on *SearchAgentsno* search agents. The operations include checking boundary conditions and calculating the objective function value *fitness* = *fobj*(Positions(i,:)), which has a time complexity of O(*dim*) because the objective function is computed for each position dimension. The leader update compares the objective function values of each search agent, which has a time complexity of O(1). Therefore, the overall objective function calculation time complexity is O(Maxiter* SearchAgentsno* *dim*);
- In each iteration, the positions of all search agents are updated based on certain mathematical formulas. Each search agent needs to traverse *dim* dimensions, so the time complexity for updating each agent’s position is O(*dim*). Each position update involves multiple formulas (e.g., updating *A*, *C*, *D*), most of which have a time complexity of O(1). Therefore, the time complexity for position updates is O(SearchAgentsno* *dim*).

**Algorithm 2.** GWOA



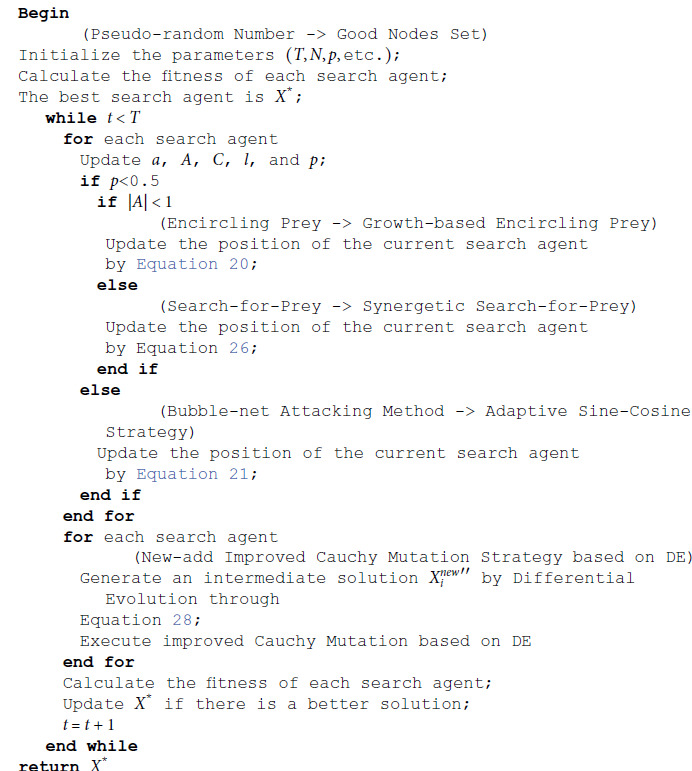



Thus, the overall time complexity of WOA is O(Maxiter* SearchAgentsno* *dim*).

GWOA introduces some improvements compared to WOA, but its time complexity is similar to WOA.

*Time Complexity of GWOA:*

- The initialization process is the same as in WOA, with a time complexity of O(SearchAgentsno* *dim*);
- The objective function calculation follows the same procedure as WOA. The outer loop runs *Maxiter* times, and the inner loop iterates over *SearchAgentsno* search agents. The time complexity for calculating the objective function and updating the leader is O(*dim*) for each agent;
- GWOA uses more complex strategies, including inertia weight updates, cosine-sine strategies, and multi-differential Cauchy mutation strategies. These increase the computational effort, and each update involves operations over *dim* dimensions, which has a time complexity of O(*dim*).

Therefore, the time complexity of GWOA is O(Maxiter* SearchAgentsno* *dim*) + O(Maxiter* SearchAgentsno* *dim*). The total time complexity is still O(Maxiter* SearchAgentsno* *dim*), but due to additional operations (such as Cauchy mutation and cosine-sine strategies), the actual computational effort is slightly higher.

#### 4.7.2 Space complexity analysis

The space complexity is primarily determined by storing the search agent position matrix and other variables.

*Space Complexity of WOA:*

- The position matrix *Positions* stores the position data of size SearchAgentsno* *dim*, with a space complexity of O(SearchAgentsno* *dim*);
- The leader’s position and score only require storing one-dimensional leader information, and the space complexity is O(*dim*). Thus, the space complexity of WOA is O(SearchAgentsno* *dim*).

*Space Complexity of GWOA:*

- GWOA stores the position matrix *Positions* the same as WOA, with a space complexity of O(SearchAgentsno* *dim*);
- Additionally, GWOA introduces extra variables (such as cosine-sine strategies and Cauchy mutation) to store intermediate values like *E*, *r*1, *r*2, etc. While these variables require less space, they still contribute to additional space consumption. The space complexity for these variables is O(SearchAgentsno* *dim*). Thus, the space complexity of GWOA is O(SearchAgentsno* *dim*).

Both WOA and GWOA have a space complexity of O(SearchAgentsno* *dim*) because they both need to store the position matrix of the search agents. GWOA also needs to store some additional intermediate variables, but their space requirements are minimal. Therefore, the space complexity of GWOA is the same as that of WOA.

Moreover, when analyzing WOA and GWOA, it is observed that the time complexity of both algorithms is generally the same. Both of them depend on the number of search agents, the problem dimension, and the number of iterations. Although GWOA improve convergence speed through strategy enhancements, its time complexity does not fundamentally change. The space complexity is directly related to the number of search agents and the problem dimension, and the complexities of the algorithms are generally the same. In most engineering design challenges, the additional computational complexity is minimal and can be ignored.

## 5 Experiments

To verify the performance and effectiveness of the GWOA algorithm, this paper uses 23 classical benchmark functions for testing. As shown in Table 1, the benchmark set includes functions F1 to F23, which are primarily used to test and evaluate the optimization algorithm’s performance under unimodal, multimodal, and compositional functions [36]. Among these, F1-F7 are unimodal functions, F8-F15 are multimodal functions, and F16-F23 are compositional functions. Each function tests different algorithm characteristics. The experiments set up in this paper are as follows, with engineering design optimization experiments presented in Chapter 6. The hardware environment for the experiment is shown in Table 2 below:

**Table 1:** Standard benchmark functions.

Function	Function’s Name	Type	Dimension (Dim)	Best Value
F1	Sphere	Uni-modal, Scalable	30/50/100	0
F2	Schwefel’s Problem 2.22	Uni-modal, Scalable	30/50/100	0
F3	Schwefel’s Problem 1.2	Uni-modal, Scalable	30/50/100	0
F4	Schwefel’s Problem 2.21	Uni-modal, Scalable	30/50/100	0
F5	Generalized Rosenbrock’s Function	Uni-modal, Scalable	30/50/100	0
F6	Step Function	Uni-modal, Scalable	30/50/100	0
F7	Quartic Function	Uni-modal, Scalable	30/50/100	0
F8	Generalized Schwefel’s Function	Multi-modal, Scalable	30/50/100	-418.98·Dim
F9	Generalized Rastrigin’s Function	Multi-modal, Scalable	30/50/100	0
F10	Ackley’s Function	Multi-modal, Scalable	30/50/100	0
F11	Generalized Griewank’s Function	Multi-modal, Scalable	30/50/100	0
F12	Generalized Penalized Function 1	Multi-modal, Scalable	30/50/100	0
F13	Generalized Penalized Function 2	Multi-modal, Scalable	30/50/100	0
F14	Shekel’s Foxholes Function	Multi-modal, Unscalable	2	0.998
F15	Kowalik’s Function	Composition, Unscalable	4	0.0003075
F16	Six-Hump Camel-Back Function	Composition, Unscalable	2	-1.0316
F17	Branin Function	Composition, Unscalable	2	0.398
F18	Goldstein-Price Function	Composition, Unscalable	2	3
F19	Hartman’s Function 1	Composition, Unscalable	3	-3.8628
F20	Hartman’s Function 2	Composition, Unscalable	6	-3.32
F21	Shekel’s Function 1	Composition, Unscalable	4	-10.1532
F22	Shekel’s Function 2	Composition, Unscalable	4	-10.4029
F23	Shekel’s Function 3	Composition, Unscalable	4	-10.5364

**Table 2:** Running environment.

Environments	Model
OS	Windows 10 22H2
CPU	AMD Ryzen7 5800H
RAM	DDR4 16G 3200Mhz
Platform	MATLAB R2024a

- Perform an ablation study by removing five improvement strategies from GWOA and testing them on selected benchmark functions;
- Perform qualitative analysis experiment on the benchmark functions for GWOA;
- Test GWOA, basic metaheuristic algorithms, and other excellent current metaheuristic algorithms on the benchmark function;
- Testing the scalability of GWOA and other metaheuristic algorithms on the benchmark functions.

The parameter settings for each algorithm are shown in Table 3:

**Table 3:** Parameter settings for metaheuristic algorithm.

Algorithm	Parameters	Value
DBO	*P*_*percent*_	0.2
	*R*	1 decreasing to 0
GWO	*a*	2 decreasing to 0
HHO	*Threshold*	0.5
SCA	*a*	2
WOA	*a*	2 decreasing to 0
	*b*	1
eWOA	*beta*	Increase from 1 to 0
	*b*	1
MSWOA	*u*	4
	*b*	1
	*z*	0.152
	*w*	Increase from 0.5 to 4
MWOA	*b*	1
	*StepfactorCF*1	2.5
	*StepfactorCF*2	1.5
AROA	*Stepfactorfr*1	0.15
	*Stepfactorfr*2	0.6
	*attractionfactorp*1	0.2
	*SearchFactorp*2	0.8
	*ExpansionfactorEf*	0.4
	*thresholdstr*1	0.9
	*thresholdstr*2	0.85
	*thresholdstr*3	0.9
ISCSO	*S*	2
	*rg*	Decrease from 2 to 0
GWOA	*a*	2 decreasing to 0
	*a*_1_	-1 decreasing to -2
	*b*	1
	ω	0.9 decreasing to 0

### 5.1 Ablation study

**Fig 3:**
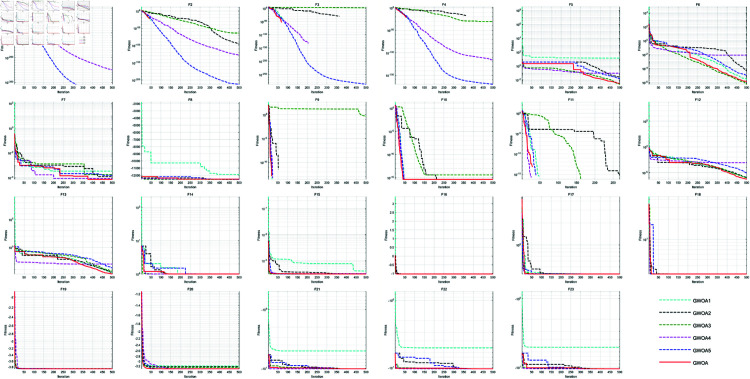
Iteration curves in ablation study.

In this section, we remove five improvement strategies from GWOA: GWOA without the Good Nodes Set initialization is named GWOA1; GWOA with the Growth-based Prey Encirclement Strategy replaced by the original WOA prey encirclement mechanism is named GWOA2; GWOA with the Synergetic Search-for-Prey strategy replaced by the original WOA prey search strategy is named GWOA3; GWOA with the Adaptive Sine-Cosine (ASC) strategy replaced by the original WOA spiral updating is named GWOA4; and GWOA without the Differential Evolution-based Enhanced Cauchy Mutation strategy is named GWOA5. The uniform settings include the maximum iteration *T*=500 and population size *N*=30. Each algorithm runs 30 times on 23 benchmark functions for performance analysis. The iteration curves are shown as Fig 3.

From the figure, it is observed that the initialization of the Good Nodes Set generates a uniformly distributed whale population. This uniform distribution helps algorithm explore the solution space more effectively and quickly, overcoming the problem of poor optimization efficiency that may arise from improper initial population selection in WOA. As a result, the convergence speed and accuracy are improved on functions F5, F15, and F21-F23. The Growth-based Encircling Prey strategy enhances the dynamic balance between exploration and exploitation in the algorithm, overcoming the issues of local optima and slow convergence speed that may occur with fixed parameter settings in WOA. In particular, it focuses more on local exploitation in the later stages, which is advantageous when handling functions F1-F6, F9-F11. The Synergetic Search-for-Prey strategy increases dependence on the optimal solution and introduces randomness to provide more search directions for the whale individuals. This improvement reduces the insufficient exploration problem that may arise in WOA for complex problems. The random perturbation further enhances the global search ability of the algorithm, demonstrating better performance on functions F1-F4, F7, F10-F11. The Adaptive Sine-Cosine (ASC) strategy considers the distance between the whale’s current position and the current best solution, thereby strengthening the exploration ability in the early stages. This strategy effectively reduces the risk of WOA getting trapped in local optima in high-dimensional and complex optimization problems, improving the global search capability. It enables the algorithm to converge quickly to the optimal solution for functions like F1-F6, F12. From F1-F6, F21-F22, the improved Cauchy Mutation strategy based on DE introduces a new perturbation mechanism, effectively helping the whale individuals escape from the current local optimal area and avoid early convergence to suboptimal solutions. This strategy helps the algorithm maintain population diversity during the search process, preventing the solution aggregation phenomenon that may occur in WOA in some cases.

Through these improvement strategies, GWOA shows significant improvements in global search ability, convergence speed, and solution accuracy compared to the traditional WOA, making it more effective in solving complex multi-constrained optimization problems.

### 5.2 Qualitative analysis experiment

**Fig 4:**
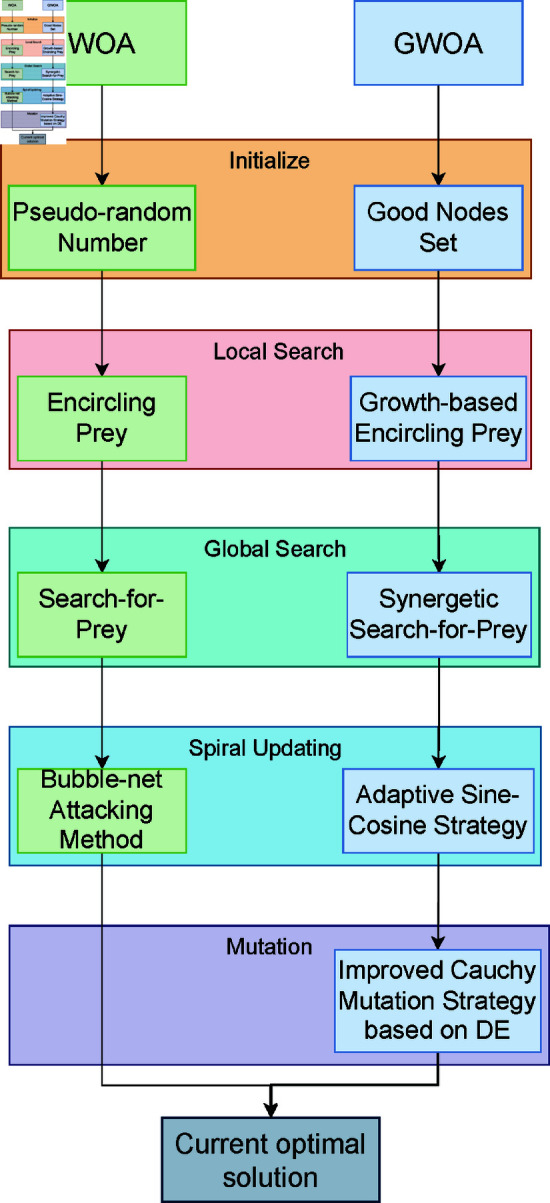
GWOA vs WOA flowchart.

**Fig 5:**
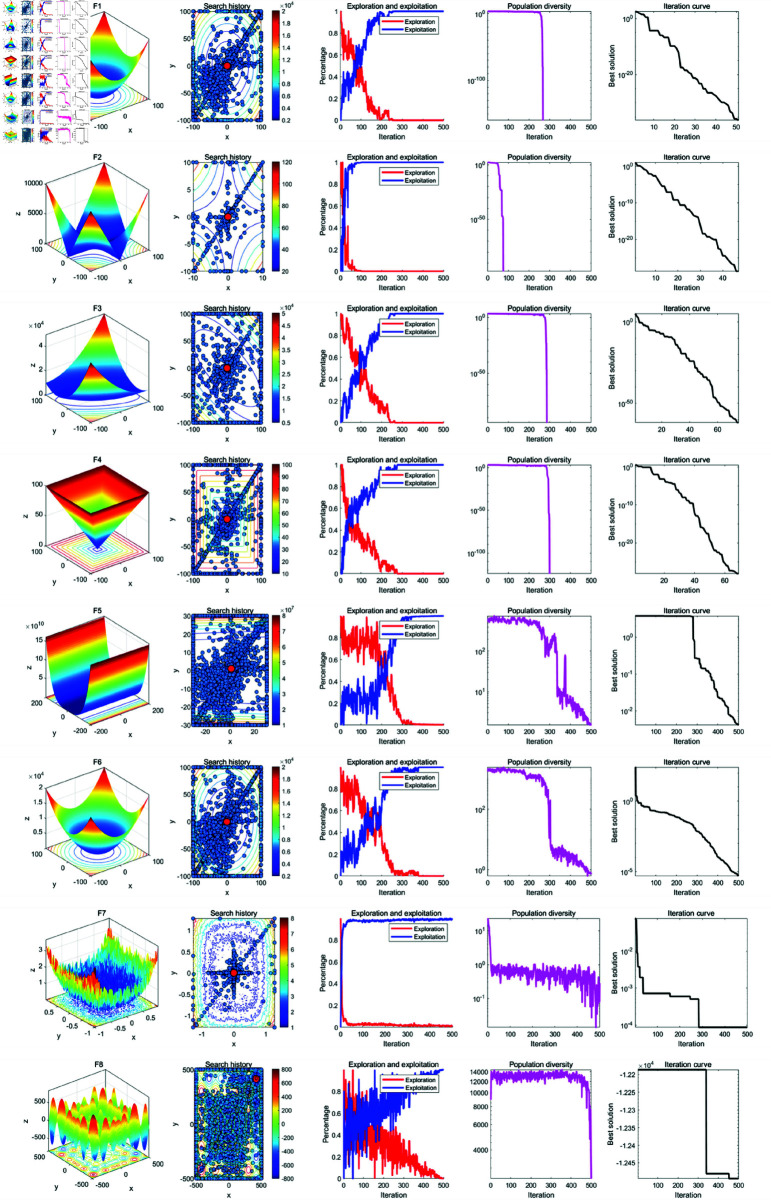
Results of the GWOA qualitative analysis (F1-F8).

**Fig 6:**
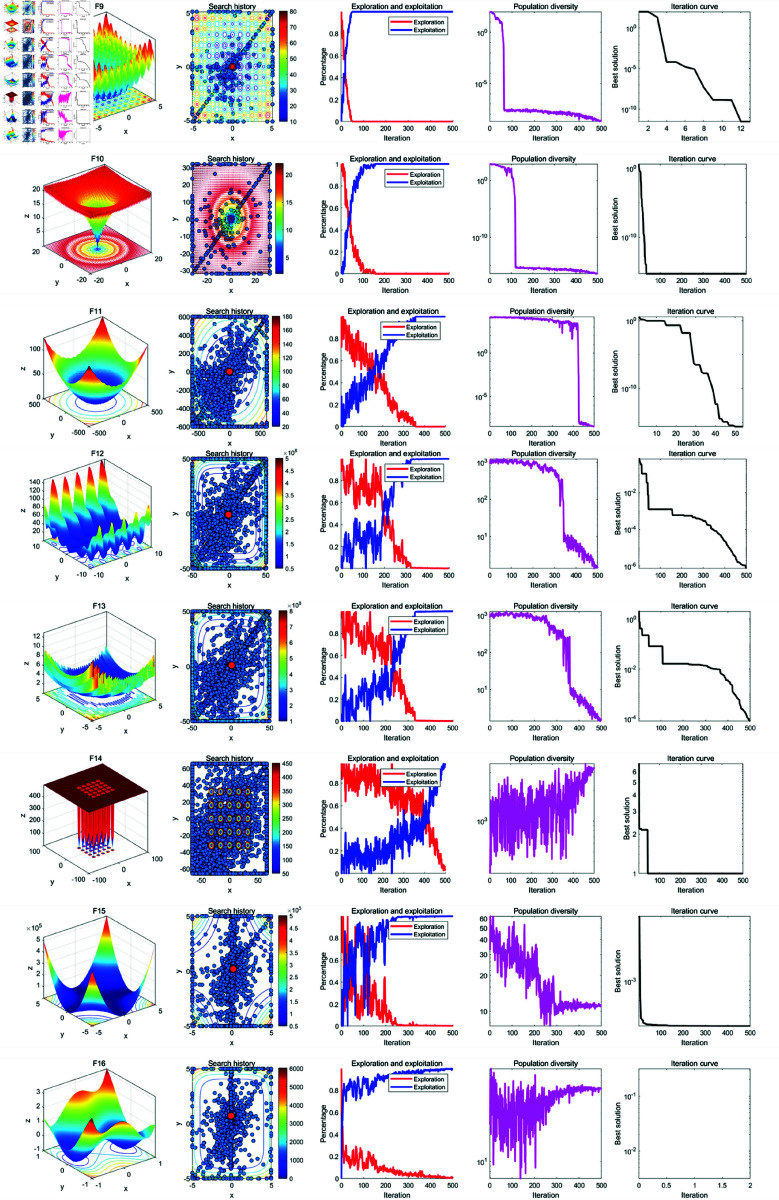
Results of the GWOA qualitative analysis (F9-F18).

**Fig 7:**
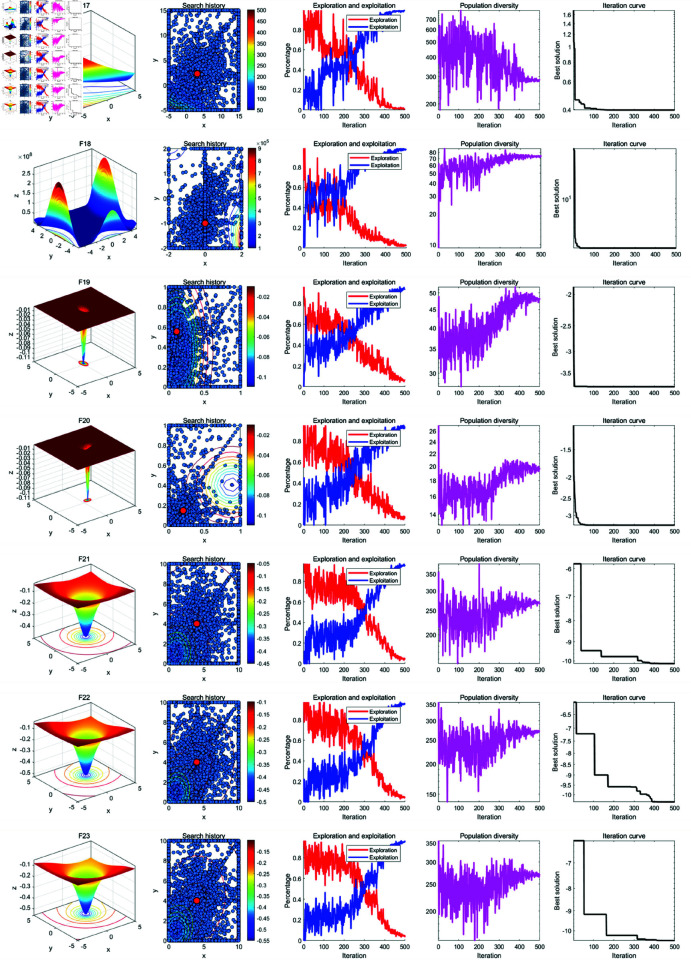
Results of the GWOA qualitative analysis (F17-f23).

In the qualitative analysis, we record the individual search history of GWOA on benchmark functions, the exploration-exploitation ratio during the iteration process, and population diversity. This allows us to comprehensively evaluate the improvement effects of GWOA. The improvement points of GWOA over WOA can be summarized in the following Fig 4. The experimental setup includes a maximum iteration count of *T*=500, function dimension *Dim*=30 and a population size of *N*=30. The results of the experiment are shown in the Fig 5, Fig 6, Fig 7 below:

From the population search history in the figure, it can be observed that GWOA, benefiting from the Good Points Set strategy, explores most of the area in unimodal functions F1-F7 and quickly converges to the global optimum. In multimodal and compositional functions F8-F23, the trajectory covers multiple potential optimal areas before converging and focusing on the neighborhood of the optimal solution. This demonstrates a good balance between global exploration and local exploitation in GWOA. In functions F20-F23, local optima were found, indicating that GWOA, when facing complex function problems, may increase computation time and converge to a local optimum.

Further analysis of the changes in the exploration-exploitation ratio during the iteration process shows that a higher exploration ratio helps escape local optima in the early stages, while a higher exploitation ratio aids in accelerating convergence in the later stages. The Growth-based Encircling Prey strategy introduces an adaptive parameter adjustment mechanism to dynamically adjust key parameters in the algorithm. As a result, in functions F5-F6, F8, F11-14, and F17-23, GWOA’s exploration-exploitation ratio exhibits a favorable state. This strategy enhances the algorithm’s search capability at different stages, with initial exploration focusing on discovery and later-stage exploitation driving convergence. Its dynamic balancing capability reflects the strong adaptability of the algorithm. In functions F1-F4, F7, F9-F10, and F15-F16, the Synergetic Search-for-Prey strategy and the Adaptive Sine-Cosine (ASC) strategy contribute to fast convergence in the early stages, demonstrating GWOA’s ability to quickly find the optimal solution. GWOA incorporates the improved Cauchy mutation strategy based on Differential Evolution to enhance search diversity. In complex functions such as F14-F23, GWOA maintains higher population diversity, preserving global search ability and avoiding premature convergence. However, in functions F1-F13, rapid convergence leads to a sharp decline in population diversity.

In summary, the performance of GWOA reflects its ability to balance exploration and exploitation, maintain diversity, and achieve convergence precision. GWOA converges quickly and stably in unimodal, functions, indicating strong exploitation capability. In multimodal and compositional functions, GWOA maintains diversity and eventually converges, demonstrating enhanced global search ability. However, in some functions, an imbalance between exploration and exploitation may lead to premature convergence.

### 5.3 Comparison of different metaheuristic algorithms

To further verify the superiority of GWOA, we selected Dung Beetle Optimization Algorithm (DBO), Grey Wolf Optimizer (GWO), Harris Hawk Optimization Algorithm (HHO), Sine Cosine Algorithm (SCA), and Whale Optimization Algorithm (WOA), An enhanced whale optimization Algorithm (eWOA) [37], Modified Whale Optimization Algorithm (MWOA) [38], a multi-strategy WOA (MSWOA) [39], Attraction Repulsion Optimization Algorithm (AROA) [40], an improved version of the sand cat swarm optimization algorithm (ISCSO) [41] as comparisons. The detailed information of the algorithm is shown in Table A1 and the detailed parameter settings of the algorithm are shown in Table A2. The algorithms are tested on selected benchmark functions with the following unified settings: maximum iteration *T*=500, population size *N*=30 and function dimension *Dim*=30. Each algorithm runs 30 times on 23 classical benchmark functions to record the average fitness (Ave), standard deviation (Std), *p*-values of Wilcoxon rank-sum test, and Friedman values for performance analysis.

#### 5.3.1 Parametric analysis.

**Fig 8:**
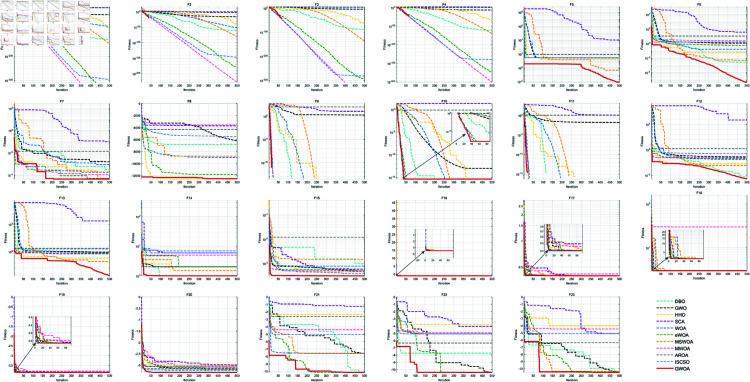
Iteration curves for comparison of different algorithms.

As shown in Fig 8 and Table 4. GWOA performs significantly better than other algorithms on F1-F7, with both the Avg and Std being 0. The Avg and Std of other algorithms on these functions are notably higher than those of GWOA. This indicates that GWOA is able to find the optimal solution and performs very stably. The convergence speed and stability of GWOA allow it to quickly find the global optimum in unimodal function problems. GWOA also shows superior performance on F9, F11-F13, and F15, suggesting that it performs well on these multimodal functions, benefiting from the Synergetic Search-for-Prey strategy and its global search ability. It is better able to escape local minima and find the global optimum. The smoothness of its curve demonstrates GWOA’s robustness and strong adaptability and consistency for different types of optimization problems. GWOA also performs excellently on F8 and F14, thanks to the application of the Good Points Set initialization. In the early stages, GWOA can quickly narrow the search space, making the solution space more regular, allowing it to find the global optimum quickly and stably. On F10 and F16-F19, the GWOA curve is similar to other algorithms because most algorithms can find very close solutions, showing minimal performance differences. On F20-F23, GWOA performs well but the results are similar to those of some other algorithms. This is because these algorithms converge to near-optimal solutions, with no significant differences. However, GWOA has a smaller Std, indicating stronger stability. By integrating various improvement strategies, GWOA possesses powerful global search ability and high stability, performing outstandingly on both unimodal and multimodal functions. In complex compositional functions, the results of GWOA are similar to those of other excellent algorithms like MSWOA and eWOA, as these algorithms can also quickly find the global optimum or near-optimal solution in these cases.

**Table 4:** Ave and Std of different algorithms.

Function	Index	DBO	GWO	HHO	SCA	WOA	eWOA	MSWOA	MWOA	AROA	ISCSO	GWOA
F1	Ave	1.4351E-112	1.4852E-27	1.0424E-65	1.8230E+01	1.0821E-72	0.0000E+00	1.0799E-147	0.0000E+00	4.8487E+00	8.6672E-283	0.0000E+00
	Std	7.6370E-112	1.4112E-27	5.7097E-65	2.4858E+01	4.5953E-72	0.0000E+00	3.8431E-147	0.0000E+00	5.2240E+00	0.0000E+00	0.0000E+00
F2	Ave	3.7784E-57	1.0087E-16	1.2390E-36	2.3513E-02	7.1296E-51	3.1347E-171	1.4896E-80	2.4107E-229	6.6533E-01	4.2663E-148	0.0000E+00
	Std	2.0629E-56	9.1618E-17	4.5310E-36	4.5197E-02	3.7644E-50	0.0000E+00	4.5070E-80	0.0000E+00	2.1417E-01	1.8615E-147	0.0000E+00
F3	Ave	5.5120E-65	4.4104E-05	4.0822E-66	8.4523E+03	4.4753E+04	6.7540E-278	3.8752E-138	0.0000E+00	1.8429E+02	2.9762E-259	0.0000E+00
	Std	3.0191E-64	1.0807E-04	1.9216E-65	6.3561E+03	1.4893E+04	0.0000E+00	6.5228E-138	0.0000E+00	2.5986E+02	0.0000E+00	0.0000E+00
F4	Ave	4.2521E-55	5.4458E-07	2.5501E-35	3.8221E+01	4.5849E+01	1.4087E-158	1.5747E-70	1.4162E-200	1.4980E+00	9.5400E-138	0.0000E+00
	Std	2.1874E-54	3.1722E-07	1.3958E-34	1.4507E+01	2.9568E+01	7.7148E-158	1.5336E-70	0.0000E+00	9.4055E-01	3.1272E-137	0.0000E+00
F5	Ave	2.5761E+01	2.6915E+01	1.2678E+01	1.4125E+05	2.8048E+01	2.8301E+01	5.7654E+00	2.8715E+01	6.5438E+01	2.7787E+01	1.8627E-02
	Std	1.8667E-01	5.7733E-01	1.4260E+01	3.4270E+05	4.7618E-01	2.9103E-01	1.1533E+01	9.5075E-02	2.1221E+01	7.7331E-01	2.0075E-02
F6	Ave	9.1804E-03	6.6285E-01	1.0964E-01	8.9296E+00	3.8330E-01	3.1325E-01	1.3064E-03	1.4763E+00	1.1611E+01	1.9795E+00	3.8882E-06
	Std	4.2941E-02	2.7558E-01	2.1859E-01	7.0620E+00	3.0024E-01	1.3697E-01	2.1641E-03	4.2666E-01	5.8475E+00	5.8545E-01	2.2803E-06
F7	Ave	1.0788E-03	2.4082E-03	1.2631E-04	1.6072E-01	4.3396E-03	8.3281E-05	1.6114E-04	5.3932E-05	3.1841E-02	1.2239E-04	1.2505E-04
	Std	8.7980E-04	1.0614E-03	8.1159E-05	2.2691E-01	4.7510E-03	8.1090E-05	1.8501E-04	4.8533E-05	3.5461E-02	1.4983E-04	7.5432E-05
F8	Ave	-9.1021E+03	-6.1716E+03	-1.2569E+04	-3.6766E+03	-1.0817E+04	-1.1689E+04	-9.2867E+03	-4.6495E+03	-4.6826E+03	-6.7875E+03	-1.2569E+04
	Std	2.1121E+03	6.4932E+02	2.2991E-01	3.1184E+02	1.6605E+03	9.9430E+02	1.4602E+03	2.0930E+03	6.9756E+02	8.3017E+02	5.8050E-02
F9	Ave	3.6279E+00	3.6774E+00	0.0000E+00	4.4928E+01	0.0000E+00	0.0000E+00	6.2885E-02	0.0000E+00	7.2135E+01	0.0000E+00	0.0000E+00
	Std	1.3086E+01	5.3800E+00	0.0000E+00	3.5417E+01	0.0000E+00	0.0000E+00	3.4443E-01	0.0000E+00	7.2109E+01	0.0000E+00	0.0000E+00
F10	Ave	4.4409E-16	1.0051E-13	4.4409E-16	1.2031E+01	4.2337E-15	4.4409E-16	4.4409E-16	4.4409E-16	7.8466E-01	4.4409E-16	4.4409E-16
	Std	0.0000E+00	1.6243E-14	0.0000E+00	9.3698E+00	2.4567E-15	0.0000E+00	0.0000E+00	0.0000E+00	3.4439E-01	0.0000E+00	0.0000E+00
F11	Ave	0.0000E+00	5.1093E-03	0.0000E+00	8.5901E-01	3.4356E-03	0.0000E+00	0.0000E+00	0.0000E+00	9.8553E-01	0.0000E+00	0.0000E+00
	Std	0.0000E+00	8.0405E-03	0.0000E+00	2.8017E-01	1.8817E-02	0.0000E+00	0.0000E+00	0.0000E+00	1.1566E-01	0.0000E+00	0.0000E+00
F12	Ave	6.2161E-04	4.0954E-02	3.4361E-04	8.6345E+03	2.2709E-02	1.7916E-02	1.1070E-03	1.0048E-01	1.2214E+00	1.4533E-01	2.2494E-05
	Std	1.6661E-03	3.5993E-02	7.8726E-04	3.2608E+04	1.7801E-02	8.7035E-03	4.0890E-03	5.5704E-02	3.6439E-01	4.8558E-02	1.0162E-04
F13	Ave	5.6361E-01	6.2791E-01	5.2926E-02	1.9917E+05	5.2243E-01	5.9861E-01	5.4106E-03	6.1797E-01	3.9596E+00	2.8515E+00	5.6708E-04
	Std	4.0063E-01	2.6528E-01	1.0322E-01	6.1635E+05	2.2982E-01	4.8749E-01	1.5530E-02	1.6939E-01	4.6484E-01	1.6616E-01	2.6699E-03
F14	Ave	1.3618E+00	4.0679E+00	1.8196E+00	1.6625E+00	3.2909E+00	4.1034E+00	1.7697E+00	7.7479E+00	5.0329E+00	4.2026E+00	9.9800E-01
	Std	7.5887E-01	4.0045E+00	1.8906E+00	9.4910E-01	3.2170E+00	3.8265E+00	1.2311E+00	3.8449E+00	3.8334E+00	3.7646E+00	2.3160E-10
F15	Ave	8.4016E-04	3.7046E-03	3.8773E-04	1.0076E-03	6.4405E-04	3.1921E-04	1.1107E-03	6.0927E-04	6.4492E-03	1.0180E-03	3.0830E-04
	Std	4.9705E-04	7.5777E-03	1.1897E-04	3.7368E-04	4.5193E-04	3.0453E-05	6.5260E-04	1.6364E-04	8.0469E-03	3.6543E-03	2.8462E-06
F16	Ave	-1.0316E+00	-1.0316E+00	-1.0316E+00	-1.0316E+00	-1.0316E+00	-1.0316E+00	-1.0316E+00	-9.9887E-01	-1.0312E+00	-1.0316E+00	-1.0316E+00
	Std	6.1849E-16	2.8831E-08	1.0912E-06	6.3495E-05	8.1638E-10	6.0318E-16	2.7556E-05	3.0125E-02	1.5696E-03	9.3295E-09	2.9218E-12
F17	Ave	3.9789E-01	3.9796E-01	3.9792E-01	3.9989E-01	3.9789E-01	3.9789E-01	3.9815E-01	4.2280E-01	3.9852E-01	3.9789E-01	3.9789E-01
	Std	0.0000E+00	3.9262E-04	6.2320E-05	2.5473E-03	7.1457E-06	7.1889E-10	3.3539E-04	2.9176E-02	1.7616E-03	4.4288E-06	8.4882E-08
F18	Ave	3.0000E+00	3.0001E+00	8.4817E+00	3.0001E+00	3.0001E+00	3.0000E+00	3.0469E+00	8.7925E+00	3.0011E+00	3.0000E+00	3.0000E+00
	Std	3.1217E-15	7.2871E-05	1.1156E+01	3.2985E-04	1.9294E-04	3.6191E-15	2.5506E-01	1.0071E+01	3.8955E-03	1.3347E-05	1.0575E-06
F19	Ave	-3.8625E+00	-3.8616E+00	-3.7407E+00	-3.8554E+00	-3.8589E+00	-3.8628E+00	-3.8611E+00	-3.7827E+00	-3.8542E+00	-3.8612E+00	-3.8628E+00
	Std	1.4385E-03	2.4539E-03	2.0953E-01	3.0806E-03	6.3748E-03	6.7097E-13	1.4192E-03	6.1760E-02	2.1061E-02	3.1523E-03	6.9251E-06
F20	Ave	-3.2006E+00	-3.2531E+00	-2.4927E+00	-2.7931E+00	-3.2093E+00	-3.2826E+00	-3.1144E+00	-2.9225E+00	-3.2214E+00	-3.2398E+00	-3.3099E+00
	Std	1.1060E-01	7.8823E-02	5.5079E-01	3.7736E-01	1.1076E-01	7.6202E-02	2.7142E-02	1.9565E-01	7.3507E-02	1.4685E-01	3.6946E-02
F21	Ave	-6.8486E+00	-9.2145E+00	-3.4113E+00	-2.6055E+00	-7.4274E+00	-1.0153E+01	-7.6087E+00	-4.5832E+00	-5.6944E+00	-5.2558E+00	-1.0153E+01
	Std	2.4168E+00	2.1601E+00	1.6440E+00	1.7968E+00	2.8147E+00	1.1484E-03	2.5066E+00	9.5905E-01	3.3948E+00	1.5331E+00	1.5540E-06
F22	Ave	-8.4927E+00	-1.0401E+01	-3.0590E+00	-2.9984E+00	-7.4225E+00	-1.0403E+01	-7.8029E+00	-4.8254E+00	-5.5720E+00	-5.9734E+00	-1.0403E+01
	Std	2.5170E+00	8.6492E-04	1.3244E+00	1.7133E+00	3.1032E+00	3.3323E-04	3.3291E+00	7.8798E-01	3.2990E+00	2.0144E+00	1.0909E-06
F23	Ave	-8.6113E+00	-9.8150E+00	-3.5390E+00	-3.1868E+00	-6.8973E+00	-1.0532E+01	-8.0813E+00	-4.7389E+00	-5.5246E+00	-6.2098E+00	-1.0536E+01
	Std	3.0638E+00	2.2348E+00	1.1990E+00	1.6244E+00	2.8776E+00	2.1025E-02	3.2145E+00	1.3268E+00	3.1327E+00	2.1997E+00	1.0928E-06

However, in performance evaluation of optimization algorithms, Avg and Std are often used to measure convergence and stability, but they may not directly reflect the algorithm’s superiority. Relying solely on these two metrics has some limitations in comparing different algorithms. So non-parametric statistical methods, such as Wilcoxon rank-sum test and Friedman test, are often used for more in-depth analysis and more reliable performance validation.

#### 5.3.2 Non-parametric Wilcoxon rank-sum test and non-parametric Friedman test.

The Friedman test is a non-parametric statistical method used to compare differences between three or more related samples. It serves as a non-parametric alternative to analysis of variance (ANOVA) and is applicable in situations where multiple experiments are conducted on the same dataset using different algorithms. This test can assess statistical differences between algorithms and identify whether significant differences exist. The performance of multiple algorithms across different datasets or test environments is ranked. The ranks are then summed, and the significance of the inter-group differences is determined using a chi-squared distribution. The Friedman test effectively reduces bias between samples, thereby enabling a fairer comparison of algorithms. As shown in the Table 5, there is a clear difference between GWOA and other excellent algorithms. GWOA’s average Friedman value is 2.3021, ranking first.

**Table 5:** Results of Wilcoxon rank-sum test and Friedman test for different algorithms.

Algorithm	Rank	Average Friedman Value	(+/=/−)
DBO	3	4.2268	(18/2/3)
GWO	8	6.5485	(23/0/0)
HHO	6	6.2166	(19/2/2)
SCA	11	9.7942	(22/0/1)
WOA	7	6.3869	(21/0/2)
eWOA	2	3.5144	(19/3/1)
MSWOA	5	5.5572	(19/2/2)
MWOA	9	6.8347	(17/5/1)
AROA	10	9.0681	(22/0/1)
ISCSO	4	5.5499	(19/3/1)
GWOA	1	2.3021	-

The Wilcoxon rank-sum test is a non-parametric statistical method designed to assess whether there are significant differences in the distributions of two independent samples. The core idea is to merge the two datasets, rank them, and calculate the statistic based on the ranks to infer distribution differences. Unlike traditional methods that rely on mean and variance, the Wilcoxon test is not affected by outliers, providing more robust results, particularly when handling non-normally distributed data. In the comparison of optimization algorithms, the Wilcoxon test is an effective tool for determining whether there is a real difference in the performance of two algorithms. If the p-value of the test is less than the preset significance level (typically 0.05), the performance difference between the two algorithms can be considered statistically significant, rather than due to random fluctuations. As shown in the Table 5 below, “+” represents a significant difference, “=” indicates equal results, and “–” denotes no obvious difference. GWOA shows significant differences compared to most algorithms, while there are more ties when compared to the excellent variant MWOA, as both ultimately converge to the optimal solution.

### 5.4 Scalability Comparison Experiment on Different Metaheuristic Algorithms

In the benchmark functions, F1-F13 are expandable functions. To explore the performance of GWOA in different dimensions, the experiments extend F1-F13 to 50 and 100 dimensions for further analysis. The algorithm’s parameter settings remain unchanged, with a maximum number of iterations set to *T*=500 and a population size of *N*=30. Then, the algorithm is run 30 times on each function, and the *p*-values of the Wilcoxon rank-sum test and the Friedman value are recorded. The experimental results are shown in Table 6:

**Table 6:** Wilcoxon Rank-sum Test and Friedman Test Results for Different Algorithms in 50 and 100 Dimensions.

Dimension	Algorithm	Rank	Average Friedman Value	(+/=/−)
Dim=50	DBO	3	4.3985	(18/4/1)
	GWO	7	6.3840	(23/0/0)
	HHO	6	6.0990	(19/3/1)
	SCA	11	9.8768	(23/0/0)
	WOA	8	6.4536	(21/0/2)
	eWOA	2	3.4166	(17/4/2)
	MSWOA	4	5.4246	(19/2/2)
	MWOA	9	6.8084	(17/5/1)
	AROA	10	8.8971	(23/0/0)
	ISCSO	5	5.6702	(19/3/1)
	GWOA	1	2.3579	-
Dim=100	DBO	3	4.4985	(17/1/5)
	GWO	9	6.9318	(23/0/0)
	HHO	6	5.9666	(17/3/3)
	SCA	11	9.9898	(23/0/0)
	WOA	7	6.3898	(21/2/0)
	eWOA	2	3.3173	(17/4/2)
	MSWOA	4	5.3579	(18/3/2)
	MWOA	8	6.5434	(18/4/1)
	AROA	10	8.9	(23/0/0)
	ISCSO	5	5.6637	(19/3/1)
	GWOA	1	2.4405	-

The results show that GWOA maintains good performance in high-dimensional environments, consistently ranking first in the Friedman Rank. In the Wilcoxon rank-sum test, it exhibits significant differences compared to other comparative algorithms. This is sufficient to demonstrate that GWOA has a strong competitive advantage over other optimization algorithms.

### 5.5 Overall effectiveness of GWOA

To further validate the performance of GWOA, this study uses a useful metric called Overall Efficiency (OE) [42] to summarize the performance results of GWOA and other algorithms. In the Table 7 below, *w* denotes win, *t* denotes tie, and *l* denotes loss. The OE of each algorithm is calculated using the following formula Eq 32:

$$OE=\frac{N-L}{L}\cdot 100\%$$ (32)

where *N* is the total number of tests; *L* is the total number of failed tests for each algorithm.

**Table 7:** Overall effectiveness of GWOA and other algorithms.

Algorithm	Dim=30 (w/t/l)	Dim=50 (w/t/l)	Dim=100 (w/t/l)	Total (w/t/l)	OE
DBO	3/2/18	4/1/18	5/1/17	12/3/53	23.18%
GWO	0/0/23	0/0/23	0/0/23	0/0/69	0%
HHO	2/2/19	1/3/19	3/3/17	6/8/55	20.28%
SCA	1/0/22	0/0/23	0/0/23	1/0/68	1.44%
WOA	2/0/21	2/0/21	0/2/21	4/2/63	8.69%
eWOA	1/3/19	2/4/17	2/4/17	5/11/53	23.18%
MSWOA	2/2/19	2/2/19	2/3/18	6/7/56	18.84%
MWOA	1/5/17	1/5/17	1/4/18	3/14/52	10.14%
AROA	1/0/22	0/0/23	0/0/23	1/0/68	1.44%
ISCSO	1/3/19	1/3/19	1/3/19	3/9/57	17.39%
GWOA	14/3/6	13/4/6	13/3/7	40/10/19	74.46%

Table 7 summarizes the performance results of GWOA and other algorithms. The results show that although the performance of GWOA decreases at high dimensions, its overall efficiency is 74.46%, making it the most effective algorithm. GWOA demonstrates excellent performance on classical benchmark functions and shows significant differences from the selected comparison algorithms. This quantitatively validate GWOA’s effectiveness

## 6 Engineering design optimization

Optimization algorithms play a key role in solving complex design and decision-making problems in engineering [43] [44]. Their objectives usually include minimizing costs, maximizing performance, or improving efficiency. These algorithms offer effective solutions, particularly in design, planning, and scheduling, contributing to efficient, economical, and sustainable system designs. This paper evaluates the performance of GWOA in engineering optimization problems through four case studies: *Pressure Vessel Design*, *Tension/Compression Spring Design*, *Piston Lever Design*, and *Speed Reducer Design*. GWOA is compared with other algorithms using a consistent setup of 500 iterations and a population size of 30. Each algorithm is run 30 times on the design problems, and performance is analyzed based on the average (Avg) and standard deviation (Std).

We applied the Penalty Function Method to manage optimization constraints. The Penalty Function Method is a widely recognized and effective technique for constraint handling. This method converts constrained optimization problems into unconstrained ones by adding a penalty term to the objective function. When a variable violates a constraint, the penalty function imposes a significant penalty. This encourages the algorithm to favor solutions that satisfy the constraints, simplifying the optimization process.

### 6.1 Pressure vessel design

**Fig 9:**
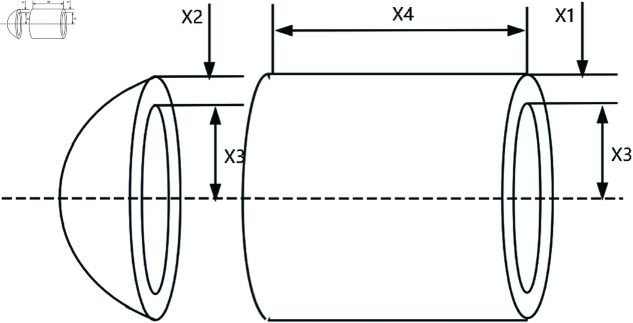
The structure of a pressure vessel.

The Pressure Vessel design problem is a classic engineering optimization problem, where the goal is to minimize the manufacturing cost of a pressure vessel. *x*_1_, *x*_2_, *x*_3_ and *x*_4_ are the design parameters representing the thickness of the vessel shell, thickness of the vessel head, inner diameter of the vessel, and length of the vessel (excluding the head) respectively. The structure of a pressure vessel as shown in the Fig 9. And the Pressure Vessel design problem is modeled as follows.

*Variable:*


$$x=[x_{1},x_{2},x_{3},x_{4}]$$

*Objective function:*

$$f(x)=0.6224x_{1}\cdot x_{3}\cdot x_{4}+1.7781x_{2}\cdot x_{3}^{2}+3.1661x_{1}^{2}\cdot x_{4}+19.84x_{1}^{2}\cdot x_{3}+punish$$ (33)

*Subject to:*

$$g_{1}(x)=-x_{1}+0.0193x_{3}\leq 0;$$ (34)

$$g_{2}(x)=-x_{2}+0.00954x_{3}\leq 0;$$ (35)

$$g_{3}(x)=-\pi x_{3}^{2}\cdot x_{4}-\frac{4}{3}\pi x_{3}^{2}+1296000\leq 0;$$ (36)

$$g_{4}(x)=x_{4}-240\leq 0;$$ (37)

*Variable range:*


$$0\leq x_{1}\leq 99;\;0\leq x_{2}\leq 99;\;10\leq x_{3}\leq 200;\;10\leq x_{4}\leq 99;$$

*Where:*

$$punish=10^{3}\cdot \sum_{i=1}^{4}\max(0,g(i){)}^{2}$$ (38)

The objective function is subject to individual constraints, *g*1 is the constraint on the ratio between the thickness and the radius; *g*2 represents the linear relationship between the inner diameter and the radius; *g*3 corresponds to the volume requirement for the container design; *g*4 is the length requirement for the container design.

In practice, the selection of suitable materials is often constrained by suppliers, cost, and physical properties such as corrosion resistance and thermal stability. Additionally, the manufacturing process of pressure vessels is complex and may involve processes such as welding and casting, which present challenges for design accuracy and cost.

### 6.2 Tension/compression spring design

In the Tension/Compression Spring design problem, the goal is to find the optimal spring parameter combination that meets performance requirements, typically minimizing the spring’s volume or weight. The Tension/Compression Spring design as shown in the Fig 11. *x*_1_ is the spring wire diameter ranging from 0.05 to 2.0, controlling the spring’s strength and stiffness. *x*_2_ is the spring outer diameter, ranging from 0.25 to 1.3, determining the spring’s spatial size. *x*_3_ is the number of active coils, ranging from 2.0 to 15.0, influencing the spring’s deformation ability. The Tension/Compression Spring design is modeled as follows.

*Variable:*


$$x=[x_{1},x_{2},x_{3},]$$

*Objective function:*

$$f(x)=(x_{3}+2)x_{2}x_{1}+punish$$ (39)

*Subject to:*

$$g_{1}=1-\frac{x_{2}^{3}x_{3}}{71785x_{1}^{4}}\leq 0;$$ (40)

$$g_{2}=\frac{\left(4x_{2}^{2}-x_{1}x_{2}\right)}{12566\left(x_{2}x_{1}^{3}-x_{1}^{4}\right)}+\frac{1}{5108x_{1}^{2}}-1\leq 0;$$ (41)

$$g_{3}=1-\frac{140.45x_{1}}{x_{2}^{2}x_{3}}\leq 0;$$ (42)

$$g_{4}=\frac{\left(x_{1}+x_{2}\right)}{1.5}-1\leq 0$$ (43)

*Variable range:*


$$0.05\leq x_{1}\leq 2;\;0.05\leq x_{2}\leq 1.3;\;2\leq x_{3}\leq 15;$$

*Where:*

$$punish=10^{3}\cdot \sum_{i=1}^{4}\max(0,g(i){)}^{2}$$ (44)

where *g*1 represents the design requirements for the stress and dimensions of the spring material; *g*2 is the optimized design of the spring’s material strength and dimensions, ensuring the spring’s safety and stability; *g*3 refers to the spring’s elastic modulus and response, ensuring the spring is neither too stiff nor too soft under working conditions; *g*4 pertains to the spring’s dimensional ratio, ensuring a reasonable overall size ratio of the spring.

Spring design typically requires very precise manufacturing processes, as even slight deviations can lead to performance instability. Springs may face fatigue failure issues over time, especially under high load and frequent operation conditions.

### 6.3 Piston lever design

The Piston Lever design problem revolves around optimizing the geometric parameters of the piston lever to meet design objectives and constraints while improving performance. The structure of a piston lever is shown in the Fig 13. The key variables typically involved are *x*_1_ for the horizontal distance of the lever, *x*_2_ for the vertical distance, *x*_3_ for the piston lever diameter, and *x*_4_ for the length of the piston lever. The goal is to minimize the material volume, thus reducing the piston lever’s weight and cost. The Piston Lever design problem can be described as:

*Variable:*


$$x=[x_{1},x_{2},x_{3},x_{4}]$$

*Objective function:*

$$f(x)=0.25\pi x_{3}^{2}(L_{2}-L_{1})+punish$$ (45)

*Subject to:*

$$g_{1}(x)=QL\cos\theta -RF\leq 0;$$ (46)

$$g_{2}(x)=Q(L-x_{4})-M_{\max}\leq 0;$$ (47)

$$g_{3}(x)=1.2(L_{2}-L_{1})-L_{1}\leq 0;$$ (48)

$$g_{4}(x)=\frac{x_{3}}{2}-x_{2}\leq 0;$$ (49)

*Variable range:*


$$0.05\leq x_{1}\leq 500;\;0.05\leq x_{2}\leq 500;\;0.05\leq x_{4}\leq 500;\;0.05\leq x_{3}\leq 120;$$

*where*

Q=10000; (50)

P=1500; (51)

L=240; (52)

$$M_{\max}=1.8\times 10^{6};$$ (53)

$$L_{1}=\sqrt{(x_{4}-x_{2}{)}^{2}+x_{1}^{2}};$$ (54)

$$L_{2}=\sqrt{(x_{4}\sin\theta +x_{1}{)}^{2}+(x_{2}-x_{4}\cos\theta {)}^{2}};$$ (55)

$$R=\frac{\left|-x_{4}(x_{4}\sin\theta +x_{1})+x_{1}(x_{2}-x_{4}\cos\theta )\right|}{\sqrt{(x_{4}-x_{2}{)}^{2}+x_{1}^{2}}};$$ (56)

$$F=0.25\pi Px_{3}^{2};$$ (57)

$$punish=10^{3}\cdot \sum_{i=1}^{4}\max(0,g(i){)}^{2}$$ (58)

Where *g*1 refers to the torque balance design, ensuring that the piston system generates sufficient torque to balance the external load; *g*2 is the maximum torque limitation design, which represents the maximum torque generated by the piston rod during operation; *g*3 pertains to the dimensional design requirements, ensuring the structural safety and stability of the system; *g*4 is the geometric design of the piston rod, ensuring its mechanical performance during operation.

The manufacturing precision of gears significantly impacts the overall system efficiency and noise levels, thus requiring high-precision processing techniques. The efficiency of the gear transmission system is often affected by factors such as friction, lubrication, and the quality of the gear surface, which necessitates the consideration of lubrication methods and material selection during the design process.

### 6.4 Speed reducer design

The Speed Reducer design problem aims to optimize the structural parameters of the speed reducer to minimize the system’s weight. The structure of a speed reducer is shown in Fig 15. The design involves 7 key variables: the gear diameter *x*_1_, the center distance *x*_2_, gear-related parameters *x*_3_, the diameter of the gear shaft *x*_4_, the diameter of another gear shaft *x*_5_, the thickness of the gear shaft *x*_6_, the thickness of another gear shaft *x*_7_. The Speed Reducer design problem is defined below:

*Variable:*


$$x=[x_{1},x_{2},x_{3},x_{4},x_{5},x_{6},x_{7}]$$

*Objective function:*

$$f(x)=0.7854x_{1}x_{2}^{2}\left(3.3333x_{3}^{2}+14.9334x_{3}-43.0934\right)-1.508x_{1}\left(x_{6}^{2}+x_{7}^{2}\right)\;\;+7.4777\left(x_{6}^{3}+x_{7}^{3}\right)+0.7854\left(x_{4}x_{6}^{2}+x_{5}x_{7}^{2}\right)+punish$$ (59)

*Subject to:*

$$g_{1}=\frac{27}{x_{1}x_{2}^{2}x_{3}}-1\leq 0;$$ (60)

$$g_{2}=\frac{397.5}{x_{1}x_{2}^{2}x_{3}^{2}}-1\leq 0;$$ (61)

$$g_{3}=\frac{1.93x_{4}^{3}}{x_{2}x_{6}^{4}x_{3}}-1\leq 0;$$ (62)

$$g_{4}=\frac{1.93x_{5}^{3}}{x_{2}x_{7}^{4}x_{3}}-1\leq 0;$$ (63)

$$g_{5}=\frac{\sqrt{16.91\times 10^{6}+{\left(\frac{745x_{4}}{x_{2}x_{3}}\right)}^{2}}}{110x_{6}^{3}}-1\leq 0;$$ (64)

$$g_{6}=\frac{\sqrt{157.5\times 10^{6}+{\left(\frac{745x_{4}}{x_{2}x_{3}}\right)}^{2}}}{85x_{7}^{3}}-1\leq 0;$$ (65)

$$g_{7}=\frac{x_{2}x_{3}}{40}-1\leq 0;$$ (66)

$$g_{8}=\frac{5x_{2}}{x_{1}}-1\leq 0;$$ (67)

$$g_{9}=\frac{x_{1}}{12x_{2}}-1\leq 0;$$ (68)

$$g_{10}=\frac{1.5x_{6}+1.9}{x_{4}}-1\leq 0;$$ (69)

$$g_{11}=\frac{1.1x_{7}+1.9}{x_{5}}-1\leq 0;$$ (70)

*Variable range:*


$$2.6\leq x_{1}\leq 3.6;\;0.7\leq x_{2}\leq 0.8;\;17\leq x_{3}\leq 28;\;7.3\leq x_{4}\leq 8.3;$$

$$7.3\leq x_{5}\leq 8.3;\;2.9\leq x_{6}\leq 3.9;\;5\leq x_{7}\leq 5.5;$$

*Where:*

$$punish=10^{3}\cdot \sum_{i=1}^{1}1\max(0,g(i){)}^{2}$$ (71)

where *g*1 refers to the power transmission efficiency design; *g*2 is the strength of the transmission system; *g*3 and *g*4 are the strength of the bearings and components; *g*5 and *g*6 pertain to the strength and stiffness of the shafts; *g*7 is the load capacity design; *g*8 refers to the gear size ratio design; *g*9 is the geometric ratio design of the gears; *g*10 and *g*11 are the relative size designs of the shafts and gears.

### 6.5 Results and analysis

**Fig 10:**
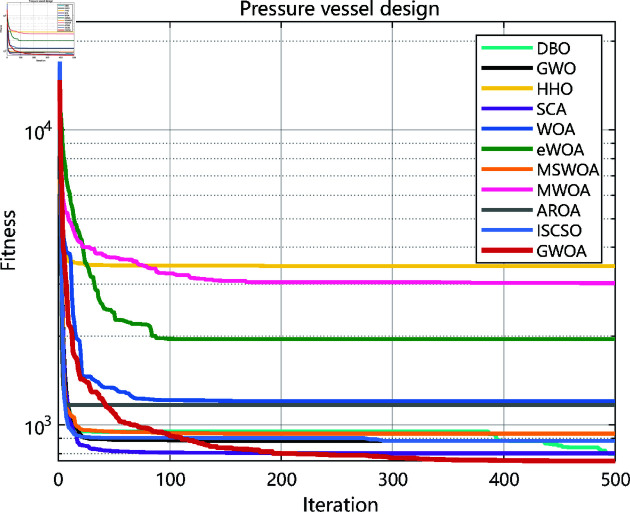
Iteration curves in pressure vessel design.

As the experimental results shown in Fig 10, Fig 12, Fig 14, Fig 16 and Table 8, GWOA outperforms other algorithms in engineering design problems by virtue of its excellent stability and optimization accuracy. It’s able to converge to the optimal solution quickly and accurately.

**Fig 11:**
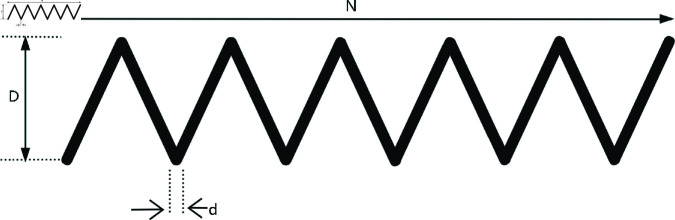
The structure of a tension/compression spring.

**Fig 12:**
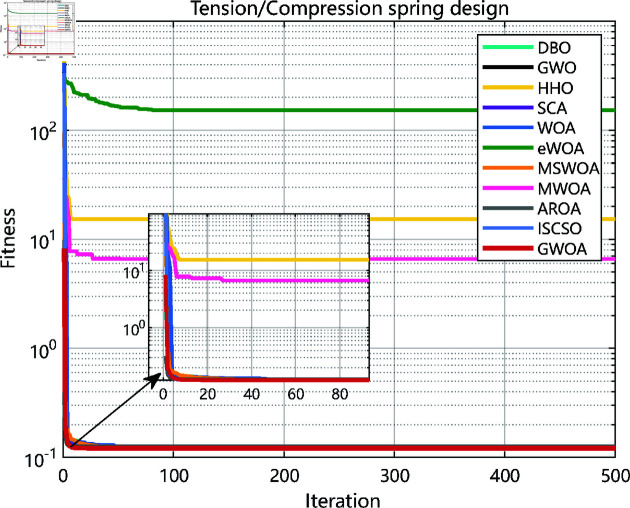
Iteration curves in tension/compression spring design.

**Fig 13:**
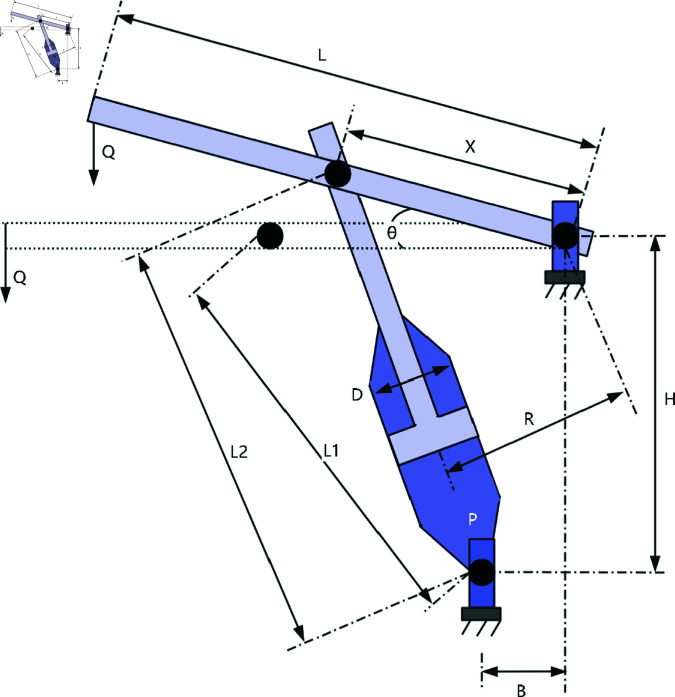
The structure of a piston lever.

**Fig 14:**
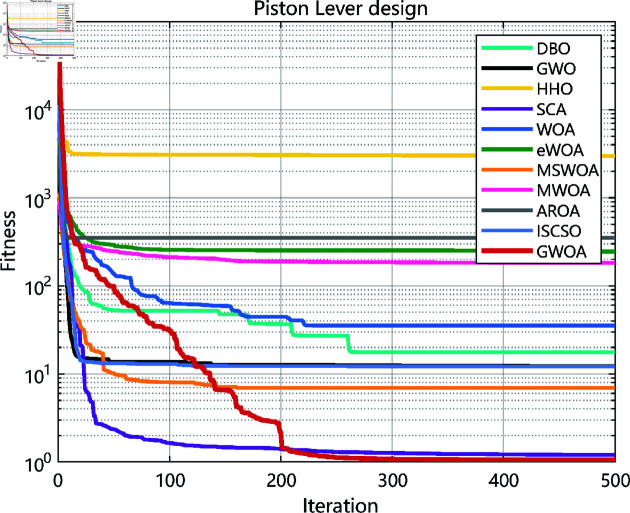
Iteration curves in piston lever design.

**Fig 15:**
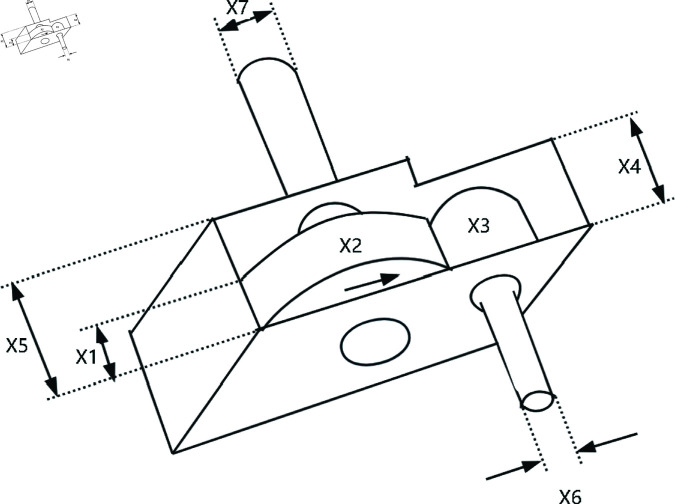
The structure of a speed reducer.

**Fig 16:**
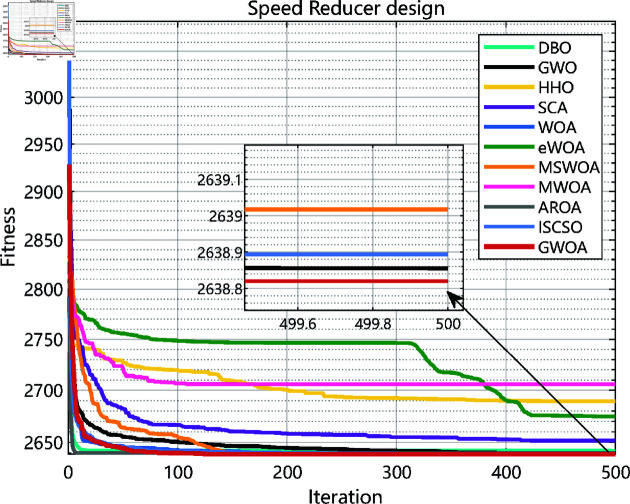
Iteration curves in speed reducer design.

**Table 8:** Results of the algorithms in solving engineering design optimization problems.

Design optimization	Index	DBO	GWO	HHO	SCA	WOA	eWOA	MSWOA	MWOA	AROA	ISCSO	GWOA
Pressure Vessel	Avg	838.73451	819.52410	1353.13785	893.89106	1230.89570	1952.348361	933.298823	3024.789724	1167.894742	881.376089	755.32370
	Std	221.01746	201.18542	259.86309	416.23736	540.82262	974.683688	433.644000	1622.873809	285.977246	260.077179	4.01738
	Best	753.52141	753.50189	936.74550	753.57467	753.50150	1095.872156	753.504380	1317.631876	753.661965	753.501436	753.52116
Tension/ Compression Spring	Avg	0.12282	0.12153	0.12152	0.12229	0.12293	145.973882	0.121550	5.760011	0.128908	0.121524	0.12152
	Std	0.00709	0.00001	0.00000	0.00085	0.00624	297.477938	0.000025	30.816079	0.022569	0.000002	0.00000
	Best	0.12152	0.12152	0.12152	0.12152	0.12152	0.121522	0.121523	0.121579	0.121522	0.121522	0.12152
Piston Lever	Avg	23.24592	28.97755	417.76391	1.28727	110.76773	244.884587	6.928540	182.174636	349.605019	12.166950	1.05765
	Std	57.53750	63.48315	330.82010	0.13255	174.93266	126.336616	31.932955	124.080196	233.108689	42.271835	0.00045
	Best	1.05718	1.05857	1.14619	1.13704	1.18361	25.739291	1.065291	12.912657	4.446859	1.057268	1.05718
Speed Reducer	Avg	2639.76094	2639.04976	2639.30171	2657.82645	2642.27419	2674.797461	2639.017224	2705.981824	2639.997778	2638.893718	2638.81983
	Std	3.97628	0.33032	1.31316	7.50618	7.84164	69.218484	0.336346	39.189790	1.791006	0.075070	0.00000
	Best	2659.51390	2640.37142	2641.72563	2670.51882	2659.51390	2659.513898	2640.724867	2787.554244	2649.028479	2639.039189	2638.81983

## 7 Conclusion

This paper proposes an improved Whale Optimization Algorithm (GWOA). First, GWOA introduces the Good Nodes Set Initialization during the initialization phase. Then, several improvement strategies are applied, including the Growth-based Encircling Prey strategy, Synergetic Search-for-Prey strategy, Adaptive Sine-Cosine strategy, and Improved Cauchy Mutation Strategy based on Differential Evolution. To validate its effectiveness, we tested GWOA using benchmark functions and engineering optimization problems. GWOA was compared with the latest improved algorithms and other classic algorithms. The experimental results show that GWOA outperforms other algorithms in terms of convergence speed, accuracy, and stability. In engineering optimization problems like pressure vessel design and spring design, GWOA also performs excellently, effectively minimizing costs while satisfying constraint conditions.

GWOA is introduced by an enhanced Cauchy Mutation based on Differential Evolution, which significantly improves its global search ability, thereby optimizing its performance in numerical optimization tasks. However, this enhancement comes with a substantial increase in computational complexity, especially when dealing with large-scale complex optimization problems. While GWOA outperforms traditional WOA in standard numerical optimization problems, the growth in computation time becomes noticeable when handling large problems. Specifically, the Differential Evolution-based enhanced Cauchy Mutation introduces additional computational steps during the exploration of the solution space, which directly impacts the algorithm’s execution efficiency. Therefore, as computational complexity increases, the feasibility of GWOA in real-time application scenarios, particularly in engineering design tasks that require fast decision-making and immediate feedback, may be limited. Since large-scale optimization problems often require results within a short time frame, the high computational cost of GWOA restricts its application in real-time optimization tasks, especially in parameter tuning tasks where rapid response times are crucial.

In summary, GWOA significantly enhances the global search capability, convergence speed, and solution accuracy of the optimization algorithm through the integration of various improvement strategies. Comparative experiments demonstrate that GWOA has advantages in solving complex, multi-constrained optimization problems, offering new insights for future engineering optimization applications. Moving forward, we plan to test the algorithm with mechanical part prototypes, verify it in real-world scenarios, and further optimize GWOA for more reliable mechanical designs. Additionally, we aim to explore GWOA’s optimization applications in other fields, such as education and healthcare, to expand its range of applicability.

## Details of the benchmark functions

To support the experimental study presented in this paper, we utilized the Standard Benchmark Functions. The relevant data have been uploaded to Figshare, and the link to the specific modeling of the Standard Benchmark Functions (*Dim*=30) is available here: https://figshare.com/s/aea70ae3f8877f7c8461. This provides reference material for further analysis by interested readers.

## Appendix Table

Table A1 is details of the metaheuristic algorithms.

**Table A1:** Details of the metaheuristic algorithms.

Algorithm	Year	Authors	Source
Dung Beetle Optimizer [5]	2022	Xue, Jiankai et al.	Inspired by the behavior of a dung beetle.
Grey Wolf Optimizer [6]	2014	Seyedali Mirjalili et al.	Inspired by the social structure and hunting behavior of the gray wolf.
Harris Hawk Optimization Algorithm [7]	2019	AA Heidari et al.	Inspired by the prey capture behavior of the Harris Hawk population.
Sine-Cosine Algorithm [35]	2016	Seyedali Mirjalili	It is inspired by the fluctuating properties of the sine and cosine functions.
Whale Optimization Algorithm [21]	2016	Seyedali Mirjalili et al	Inspired by the feeding behavior of humpback whales.
enhanced Whale Optimization Algorithm [37]	2021	Chakraborty S et al.	Enhanced search mechanism introduced
MSWOA [39]	2022	YangWenbiao et al.	Combines the SCA and the WOA
MWOA [38]	2021	Anitha J et al.	The concept of multi-verse has been adopted.
Attraction-Repulsion Optimization Algorithm [40]	2024	K Cymerys	Based on the attraction- rejection mechanism.
ISCSO [41]	2024	Niu Yanbiao et al.	Inspired by the hunting behavior of sand cats.

Table A2 are detailed parameters for engineering design problems.

**Table A2:** Details of parameter settings.

Parameters	Pressure Vessel design	Tension/Compression Spring design	Piston Lever design	Speed Reducer design
Maximum iteration T	500	500	500	500
Population size N	30	30	30	30
Dimension Dim	30	30	30	30
Run Times	30	30	30	30
lb	[0,0,0,0]	[0.05,0.25,2.00]	[0.05 0.05 0.05 0.05]	[2.6, 0.7, 17, 7.3, 7.3, 2.9, 5]
ub	[99,99,200,200]	[2,1.3,15.0]	[500 500 120 500]	[3.6, 0.8, 28, 8.3, 8.3, 3.9, 5.5]
